# Neutrophil extracellular trap-mediated impairment of meningeal lymphatic drainage exacerbates secondary hydrocephalus after intraventricular hemorrhage

**DOI:** 10.7150/thno.91653

**Published:** 2024-02-24

**Authors:** Qiang Zhang, Yujie Chen, Yingpei Li, Zhou Feng, Liang Liang, Xiaoke Hao, Wenbo Kang, Zhaoqi Zhang, Xuyang Zhang, Rong Hu, Hua Feng, Zhi Chen

**Affiliations:** 1 Department of Neurosurgery and State Key Laboratory of Trauma, Burn and Combined Injury, Southwest Hospital, Third Military Medical University (Army Medical University), Chongqing, 400038, China.; 2 Department of Neurosurgery, The 961 st Hospital of the Chinese People's Liberation Army Joint Logistic Support Force, Qiqihar, 161000, Heilongjiang Province, China.; 3 CAS Key Laboratory of Separation Science for Analytical Chemistry, Dalian Institute of Chemical Physics, Chinese Academy of Sciences, Dalian, 116023, China.; 4 Department of Rehabilitation, Southwest Hospital, Third Military Medical University (Army Medical University), Chongqing, 400038, China.; 5 Chongqing Key Laboratory of Precision Neuromedicine and Neuroregenaration, Southwest Hospital, Third Military Medical University (Army Medical University), Chongqing, 400038, China.; 6 Chongqing Clinical Research Center for Neurosurgery, Southwest Hospital, Third Military Medical University (Army Medical University), Chongqing, 400038, China.

**Keywords:** Intraventricular hemorrhage, Hydrocephalus, Meningeal lymphatic vessels, Neutrophil extracellular traps, CX3C chemokine receptor 1

## Abstract

**Rationale:** Hydrocephalus is a substantial complication after intracerebral hemorrhage (ICH) or intraventricular hemorrhage (IVH) that leads to impaired cerebrospinal fluid (CSF) circulation. Recently, brain meningeal lymphatic vessels (mLVs) were shown to serve as critical drainage pathways for CSF. Our previous studies indicated that the degradation of neutrophil extracellular traps (NETs) after ICH/IVH alleviates hydrocephalus. However, the mechanisms by which NET degradation exerts beneficial effects in hydrocephalus remain unclear.

**Methods:** A mouse model of hydrocephalus following IVH was established by infusing autologous blood into both wildtype and Cx3cr1^-/-^ mice. By studying the features and processes of the model, we investigated the contribution of mLVs and NETs to the development and progression of hydrocephalus following secondary IVH.

**Results:** This study observed the widespread presence of neutrophils, fibrin and NETs in mLVs following IVH, and the degradation of NETs alleviated hydrocephalus and brain injury. Importantly, the degradation of NETs improved CSF drainage by enhancing the recovery of lymphatic endothelial cells (LECs). Furthermore, our study showed that NETs activated the membrane protein CX3CR1 on LECs after IVH. In contrast, the repair of mLVs was promoted and the effects of hydrocephalus were ameliorated after CX3CR1 knockdown and in Cx3cr1^-/-^ mice.

**Conclusion:** Our findings indicated that mLVs participate in the development of brain injury and secondary hydrocephalus after IVH and that NETs contribute to acute LEC injury and lymphatic thrombosis. CX3CR1 is a key molecule in NET-induced LEC damage and meningeal lymphatic thrombosis, which leads to mLV dysfunction and exacerbates hydrocephalus and brain injury. NETs may be a critical target for preventing the obstruction of meningeal lymphatic drainage after IVH.

## Introduction

Spontaneous intracerebral hemorrhage (ICH) is associated with a high mortality and morbidity rate, making it a major threat to human health. The incidence of the ICH complication intraventricular hemorrhage (IVH) can reach up to 50%, and IVH and consequent hydrocephalus are significant factors contributing to poor prognosis in these patients 1. Previous studies have indicated that hemorrhage and metabolic byproducts are crucial factors that contribute to secondary damage in ICH/IVH. Currently, surgical treatment or (combined) pharmacological thrombolysis for clot liquefaction is the main strategy to alleviate secondary hydrocephalus. However, invasive CSF shunting poses a high risk of severe complications, including shunt obstruction or infection 2-5. Additionally, the results of the CLEAR III trial on intraventricular fibrinolysis for IVH have been disappointing, and the efficacy of hematoma clearance and improvements in neurological outcomes did not meet the expected targets 6. As the clearance mechanisms after IVH due to ICH or subarachnoid hemorrhage (SAH) are particularly complex, treatment strategies that promote intraventricular fibrinolysis represent double-edged swords, as they both benefit and harm IVH patients simultaneously 7. However, the timing of treatment may also be relevant for achieving successful intraventricular fibrinolysis and good patient outcomes 8. Therefore, it is necessary to develop low-risk targeted drug therapies to treat hydrocephalus patients after IVH.

Neutrophil extracellular traps (NETs), which were first reported in 2004 as a defense mechanism against pathogenic microorganisms, consist of web-like structures formed by extracellular DNA released by activated neutrophils. These structures are embellished with histones and various granule proteins. NETs play a crucial role in various pathological inflammatory injuries and thrombus formation 9-12. Recent investigations have demonstrated NET formation in arterial thrombi and the brain tissue of patients with acute cerebral ischemia 13. An increase in the plasma markers of NETs is associated with an unfavorable prognosis 14. In a preliminary study on the clearance mechanisms of hematoma after ICH/IVH, the research team found substantial infiltration of neutrophils and the formation of NETs following ICH/IVH 15. Clearing NETs accelerated hematoma removal and improved hydrocephalus 16, suggesting that NET formation could be a therapeutic target for IVH and secondary hydrocephalus, warranting further investigation.

Impairment of cerebrospinal fluid (CSF) circulation is the primary mechanism of posthemorrhagic hydrocephalus 17-19. For a long time, the exact pathways and functions of CSF circulation remained unclear. The traditional belief was that CSF primarily acted as a physical buffer and waste reservoir and was ultimately absorbed through arachnoid villi 20. However, a groundbreaking study published in Nature in 2015 by the research team led by Kipnis J revealed the presence of mLVs and established a connection between the brain and cervical lymph nodes (CLNs) 21. Brain meningeal lymphatic vessels (mLVs) were shown to possess the ability to drain CSF components and clear large molecules. This research has opened new possibilities in the study of hematoma clearance in hemorrhagic diseases and hydrocephalus. Subsequent research on subarachnoid hemorrhage (SAH) revealed that red blood cells (RBCs) swiftly returned to deep cervical lymph nodes (dCLNs) via mLVs, consequently mitigating the secondary damage induced by subarachnoid hemorrhage 22. Further studies on mice with ICH showed that impaired meningeal lymphatic function hinders hematoma absorption and exacerbates impairments 23. The involvement of mLVs in CSF clearance has also been demonstrated in rodents, humans, and nonhuman primates 24-26. The meningeal lymphatic system is a crucial drainage pathway for CSF 27. These studies suggest that the meningeal lymphatic system plays a role in clearing metabolic byproducts and CSF in hemorrhagic diseases. Regulating drainage may provide new avenues for the prevention and treatment of posthemorrhagic hydrocephalus and brain injuries.

Considering that the formation of NETs is a significant mechanism of various pathological microcirculatory disorders, we examined whether the widespread distribution of NETs in the ventricles and subarachnoid space after IVH could also cause disturbances in cerebral lymphatic circulation. A recent study showed that in mice with bacterial meningitis, NET-induced dysfunction of the meningeal lymphatic and glymphatic systems disrupted CSF circulation and absorption, and this outcome was improved by the administration of DNase I to alleviate the damage 28. Follow-up studies by our team showed that after SAH, DNase I not only improved CSF circulation but also facilitated the drainage of Evans blue (EB) dye from the cerebral ventricles to the dCLNs, suggesting that the degradation of NETs improved CSF circulation and meningeal lymphatic drainage 29. These research findings indicate that obstructing meningeal lymphatic drainage may be a crucial step in the exacerbation of secondary hydrocephalus after IVH. Furthermore, lymphatic thrombosis has gained attention in recent years. A recent study showed the formation of lymphatic thrombosis induced by NETs in the lungs and local lymph nodes of COVID-19 patients 30. Other studies have shown that infections, amyloidosis, cancer, and lymph node clearance may lead to lymphatic thrombosis, and mechanism is associated with lymphatic endothelial cell (LEC) injury 31. Thus, combining the research by our team and recent reports, we hypothesize that NETs induced by IVH result in lymphatic thrombosis, thereby obstructing meningeal lymphatic drainage, hindering the clearance of metabolic waste products and CSF, and consequently worsening secondary hydrocephalus and brain injury. This study investigated the effect of targeting NETs in the treatment of hydrocephalus to identify new therapeutic targets for hydrocephalus patients.

## Materials and Methods

### Animals

We procured a total of 351 adult male C57BL/6 mice (aged 6 to 8 weeks) (0.8% mortality) from the Experimental Animal Center of the Army Medical University. Cx3cr1^-/-^ mice (18 adult males), which were generated using CRISPR/Cas9-mediated gene editing, were purchased from Cyagen Biosciences (Suzhou, China). A reverse 12-h dark/12-h light cycle environment was used to acclimate the mice, who were also provided with unlimited access to water and food. All animal-related experimental procedures were performed according to the Guidelines for the Care and Use of Laboratory Animals, which were approved by the Laboratory Animal Welfare and Ethics Committee of Third Military Medical University (AMUWEC20232125). Moreover, the reporting of these procedures followed the ARRIVE (Animal Research: Reporting of In Vivo Experiments) guidelines.

### Experimental design

**Figure 1:**
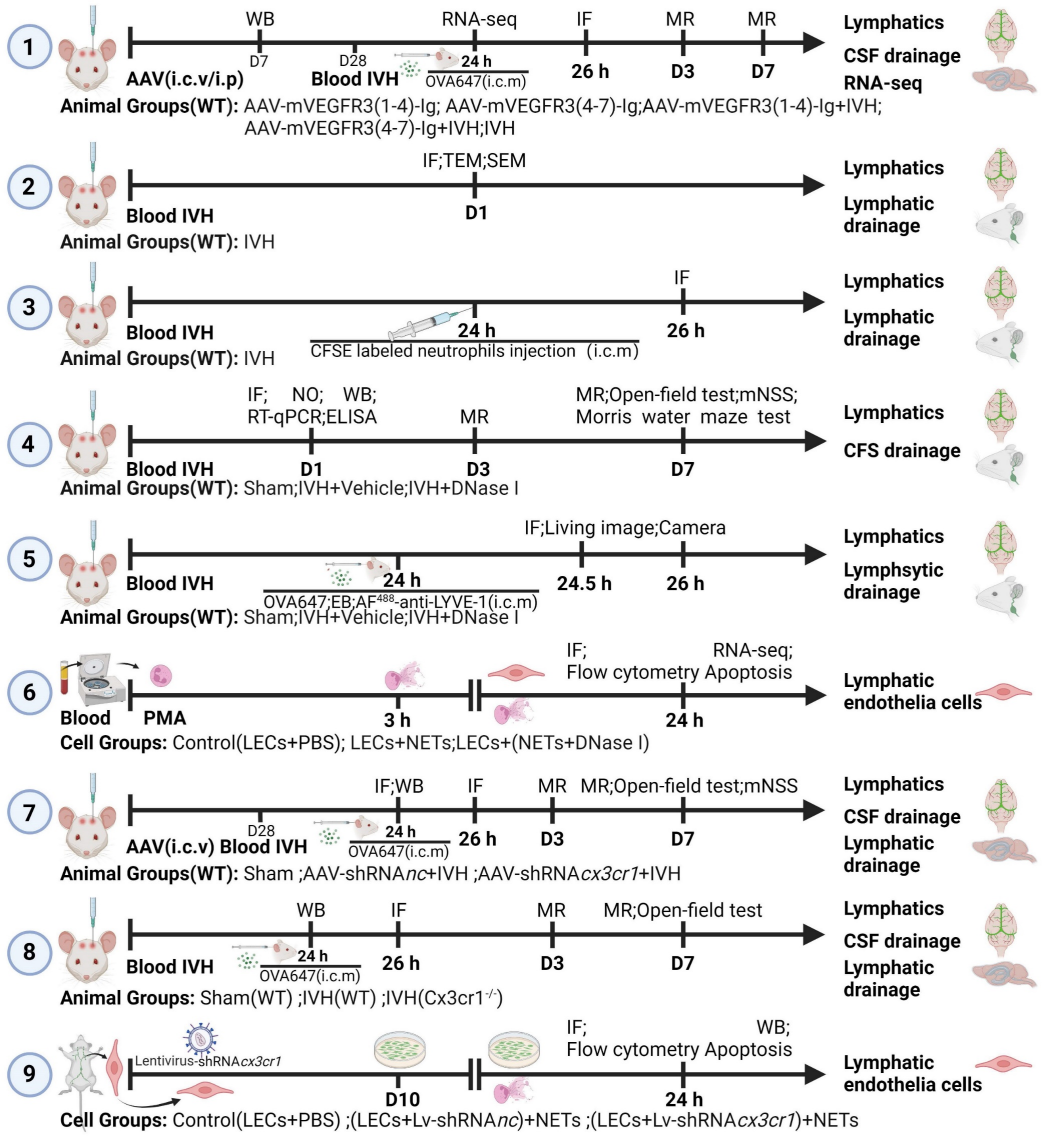
** Illustration of the experimental design and subsequent analyses in a schematic format.** i.c.v.: intracerebroventricular; i.c.m.: intracisterna magna; i.p.: intraperitoneal injection; MR: T2-weighted images; TEM: transmission electron microscopy; SEM: scanning electron microscopy; IF: immunofluorescence staining; WB: Western blot; RNA-seq: RNA sequencing; ELISA: enzyme-linked immunosorbent assay; OVA647: ovalbumin labeled with Alexa Fluor 647; EB: Evans blue; NETs: neutrophil extracellular traps.

The study included nine experiments, which were designed as follows and are shown in Figure 1.

**Experiment 1:** To investigate the impact of mLV ablation on secondary hydrocephalus and changes in mRNA expression in the intraventricular wall after IVH, 42 (6-week-old) mice were randomly assigned to five groups (n = 6): the AAV-mVEGFR3(1-4)-Ig group, AAV-mVEGFR3(4-7)-Ig group, AAV-mVEGFR3(1-4)-Ig+IVH group, AAV-mVEGFR3(4-7)-Ig+IVH group, and IVH group. The AAV-mVEGFR3(1-4)-Ig group and the AAV-mVEGFR3(1-4)-Ig+IVH group received intracerebroventricular (i.c.v.) injections of AAV-mVEGFR3(1-4)-Ig to induce mLV drainage dysfunction, while the AAV-mVEGFR3(4-7)-Ig group and the AAV-mVEGFR3(4-7)-Ig+IVH group were administered AAV-mVEGFR3(4-7)-Ig as a control 32, 33. The IVH group received an i.c.v. injection of PBS. The virus transfection efficiency was measured by Western blot (WB) analysis of serum 7 days after intraperitoneal injection (i.p.) of the virus. Four weeks later, IVH was induced with autologous blood. OVA647 was administered by intracisterna magna (i.c.m.) injection 34. Immunofluorescence (IF) staining of LYVE-1 was performed to examine the drainage of OVA647 in dCLNs. At 24 h after IVH, the ventricular wall was examined by RNA-seq analysis to determine changes in mRNA expression 35. T2-weighted images were obtained using 7.0 T small animal magnetic resonance imaging (MRI) 3 and 7 days after IVH, and the volume of the lateral ventricles was calculated using 3D Slicer software to dynamically monitor changes in ventricular volume 17.

**Experiment 2:** To observe the pathological changes in mLVs following IVH (18 mice), the meninges and dCLNs were collected 24 h after IVH. IF staining (Ly6g, CitH3, LYVE-1, fibrin) was performed on the meninges and dCLNs to examine the characteristics of neutrophils, NETs, and fibrin in mLVs and dCLNs. The microstructure of mLVs after IVH was observed by scanning electron microscopy (SEM) and transmission electron microscopy (TEM) 36.

**Experiment 3:** To investigate the drainage of neutrophils via the meningeal lymphatic system, autologous blood samples were obtained from mice via cardiac puncture. Neutrophils were isolated using gradient centrifugation and labeled with carboxyfluorescein diacetate succinimidyl ester (CFSE) 37. CFSE-labeled neutrophils were subsequently intraventricularly injected 24 h after IVH (3 mice), and the mice were euthanized 2 h post-injection 22. IF staining of the meninges was performed to examine the drainage of neutrophils via mLVs and dCLNs.

**Experiment 4:** To explore the effects of NET degradation on post-IVH hydrocephalus, neurologic deficits and mLV damage, 90 mice were randomly assigned to three groups: the Sham group, IVH+vehicle group, and IVH+DNase I group. The IVH+DNase I group received an i.c.v. injection of 300 U (2 µL) DNase I 1 h after IVH induction. Subsequent injections of DNase I (150 U/μL in 2 µL) were administered at 12-h intervals until the predetermined time points 38. For IF staining, Western blot (WB) analysis, RT‒qPCR, and enzyme-linked immunosorbent assay (ELISA), the meninges were collected 24 h after IVH. IF staining of the meninges was performed to evaluate the area of LYVE-1 staining in the confluence of sinus (COS) region and the number of TUNEL-positive cells in the mLVs to evaluate damage to LECs. WB analysis was performed to quantitatively evaluate the changes in citrullinated histone H3 (CitH3), VEGFC, LYVE-1, PROX1, FOXC2, and VE-cadherin in the meninges to investigate the correlation between NET degradation and LEC activity 34, 39. ELISA and RT‒qPCR were used to evaluate the expression of VEGFC and FOXC2 in mLVs to observe damage to mLVs after IVH 39. The levels of nitric oxide (NO) in mLVs were determined using an NO assay kit to evaluate the extent of mLV impairment after IVH 39. For MRI, DNase I treatment was administered until day 2, and secondary hydrocephalus was assessed using MRI three and seven days after IVH. An open-field test was performed to examine motor function after IVH. The Morris water maze test was performed on day 7 after IVH to evaluate memory 17. The modified Neurological Severity Score (mNSS) scale was used to assess neurological deficits.

**Experiment 5:** To investigate the influence of NET degradation on CSF tracers, 90 mice were randomly assigned to three groups: the Sham group, IVH+vehicle group, and IVH+DNase I group. At 24 h after IVH, i.c.m. injection of EB, OVA647 (fluorescence-labeled ovalbumin conjugated to Alexa Fluor 647), or AF^488^-anti-LYVE-1 was administered. Samples were collected at 0.5 or 2 h later. IF staining was performed to assess the drainage of EB and OVA647 in mLVs and dCLNs (LECs were labeled with LYVE-1) 34, 39. The fluorescence intensity of superficial cervical lymph nodes (sCLNs) was measured using a small animal in vivo fluorescence imaging system. MLVs were observed by IF staining. The percentage of AF^488^ anti-LYVE-1 (i.c.m.) relative to LYVE-1 (ex vivo) was calculated to determine the flow velocity of mLVs 22.

**Experiment 6:** To investigate the impact of NETs on primary LECs, primary LECs were assigned to three groups: the Control (LEC+PBS), LEC+NET, and LEC+(NET+DNase I) groups. Neutrophils were isolated from 18 mice using the autologous blood gradient centrifugation method. NET formation was induced by phorbol 12-myristate 13-acetate (PMA) 40, and purified NETs, as well as PBS- and DNase I-treated NETs, were cultured with primary LECs for 24 h. RNA was extracted for RNA sequencing analysis to examine differences in gene expression. Cell proliferation was assessed with the CCK-8 assay. PI^+^ cells were detected using flow cytometry. The quantification of TUNEL-positive cells was performed using immunofluorescent TUNEL staining.

**Experiment 7:** To confirm that CX3CR1 is a critical molecule in NET-induced damage to LECs in vivo, 72 mice (6 weeks old) were randomly assigned to three groups: the Sham group, AAV-shRNA*nc*+IVH group, and AAV-shRNA*cx3cr1*+IVH group. AAV-shRNA*cx3cr1* was engineered to knock down the expression of CX3CR1 in mLVs after i.c.v. injection. AAV-shRNA*nc* was injected as a control. Autologous blood IVH mice were generated 28 days after viral injection. Mouse meninges were harvested 24 h after IVH, and changes in CX3CR1 expression were assessed by IF staining and WB analysis. OVA647 was injected into the cisterna magna 24 h after IVH, and the IF intensity of OVA647 in dCLNs was observed 2 h after i.c.m. injection 34. Secondary hydrocephalus was evaluated using MRI 3 and 7 days after IVH 17. Mouse motor function was assessed using the open-field test 7 days after IVH. The mNSS scale was used to assess neurological deficits.

**Experiment 8:** To confirm that CX3CR1 is a critical molecule in NET-induced damage to LECs in vivo, 54 mice were randomly assigned to three groups: the Sham (WT) group, IVH (WT) group, and IVH (Cx3cr1^-/-^) group. Mouse meninges were harvested 24 h after IVH, and changes in CX3CR1 expression were assessed by WB analysis. The number of TUNEL-positive cells in mLVs was determined by IF staining to evaluate damage to LECs. OVA647 was injected into the cisterna magna 24 h after IVH, and the IF intensity of OVA647 in dCLNs was observed 2 h after i.c.m. injection. Secondary hydrocephalus was evaluated using MRI 3 and 7 days after IVH. Mouse motor function was assessed using the open-field test 7 days after IVH.

**Experiment 9:** To confirm that CX3CR1 is a critical molecule in NET-induced damage to LECs in vitro, primary LECs cultured in dishes were randomly assigned to three groups: the Control (LEC+PBS) group, (LEC+Lv-shRNA*nc*)+NET group, and (LEC+Lv-shRNA*cx3cr1*)+NET group. Primary LECs were transfected with the lentivirus (Lv-shRNA*cx3cr1,* Lv-shRNA*nc*). Following successful transfection, CX3CR1 knockdown efficiency was assessed by WB analysis. Cell proliferation was determined by the CCK-8 assay. Flow cytometry was used to detect PI^+^ cells. The quantification of TUNEL-positive cells was performed using immunofluorescent TUNEL staining.

### Human samples

For patients within three days of onset with ICH/IVH, 2 mL of CSF was obtained during the initial insertion of an extraventricular drain (EVD). The collection of human cerebrospinal fluid samples was approved by the Ethics Committee of the Southwest Hospital of Third Military Medical University (approval No. KY2022128), and the study was performed following the Declaration of Helsinki. Informed consent was waived for the following analysis. The samples (2 mL, CSF) were promptly placed on ice and centrifuged twice at 4 °C at 500 × g for 5 min each, after which the supernatant was frozen at -80 °C pending further analysis. The quantification of cell-free DNA (cfDNA) in CSF samples was carried out as previously described 41. In detail, the samples were incubated with the fluorescent cfDNA-binding dye Sytox Green (P11496, Thermo Fisher) at a concentration of 1 μM for 5 min. The fluorescence intensity was measured in 96-well microplates with a Tecan Infinite M200 reader at an excitation wavelength of 480 nm and an emission wavelength of 520 nm. The values obtained were then normalized against a standard curve of dsDNA (Lambda DNA, Thermo Fisher).

### Animal model of IVH

As previously described, an IVH model was established by injecting autologous blood through the lateral ventricle 42. Briefly, the mice were deeply anesthetized with 2% isoflurane and secured on a stereotaxic instrument (RWD, Shenzhen, China). Autologous blood (50 μL) was injected into the right lateral ventricle with a Hamilton microsyringe and microinfusion pump at a speed of 5 μL/min (coordinates: bregma, posterior 0.50 mm; lateral 1.50 mm; depth 2.20 mm). Following injection, the device was left in place for 5 min before the mice were transferred to a warming chamber until they regained consciousness. The Sham group were injected with an equivalent volume of saline solution. Postoperatively, the mice were allowed free access to water and solid food under constant light conditions.

### CSF tracer injection

The mice were anesthetized by i.p. injection with ketamine (80 mg/kg) and xylazine (8 mg/kg) in a saline solution. Following anesthesia, the mice were placed in a stereotaxic instrument, and the skin at the posterior neck was incised. The muscles were separated to expose the cisterna magna. To observe the drainage of the CSF tracer in the mLVs and dCLNs, 5 μL of OVA647 (2 mg mL^-1^ in PBS, O34784, Thermo Fisher) or 5 μL of EB (2%, 5 mL/kg, E2129, Sigma Aldrich) was slowly injected into the cisterna magna using a 25 μL Hamilton microsyringe. To visualize the flow velocity of mLVs, a solution containing 5 μL of Alexa Fluor 488-conjugated anti-LYVE-1 antibody (AF^488^-anti-Lyve1) (53-0443-82, Thermo Fisher) was slowly injected into the cisterna magna at a rate of 1 μL/min. To minimize the risk of backflow and leakage, the injection needle was maintained in place for 2 min. Then, the mice were sutured and placed on a 37 °C heating pad until they regained consciousness. The AF^488^-conjugated LYVE-1 antibody was allowed to circulate for 30 min, and OVA647 was allowed to circulate for 2 h, after which the mice were euthanized. To prevent the backflow of CSF, the needle was kept in place for 5 min after injection 34.

### Lymphatic ablation

The AAV virus used was serotype 9 and was provided by Shanghai GeneChem Gene Technology (GV461). AAV transduction in C57BL/6 mice was achieved through i.p. or i.c.v. injection. The mice were 6 weeks of age at the time of AAV transduction. For i.c.v. administration, 4 µL of AAV (containing 1x10^10^ viral particles) was injected at a rate of 0.5 µL/min. After injection, a 5-min pause was observed to prevent backflow and leakage. For i.p. administration, 200 µL of AAV (containing 1x10^12^ viral particles) was administered. The experimental group received AAV-mVEGFR3(1-4)-Ig, which encodes the ligand of VEGFR3 stuctural domains 1-4 fused to the IgG Fc domain, while the Control group was injected with AAV-mVEGFR3(4-7)-Ig, which encodes VEGFR3 structural domains 4-7 but does not bind to VEGF-C or VEGF-D. The autologous blood IVH model was induced 28 days after viral injection.

### CX3CR1 knockdown

The AAV virus used was serotype 9 and was provided by Shanghai GeneChem Gene Technology (GV478). AAV transduction in C57BL/6 mice was achieved by i.c.v. administration. The mice were 5-6 weeks of age at the time of AAV transduction. For i.c.v. administration, 4 µL of AAV (containing 1x10^10^ viral particles) was injected at a rate of 0.5 µL/min. After injection, a 5-min pause was observed to prevent backflow and leakage. The autologous blood IVH model was induced 28 days after viral injection. The knockdown sequences used in this study were AAV-shRNA*cx3cr1-1*: GCATATTCTTCATCACCGTCA, AAV-shRNA*cx3cr1-2*: GCAACTCGGAAGTCAACATCC, and AAV-shRNA*cx3cr1-3*: GACAGCATTCTGAGCAGTTT.

### DNase I. i.c.v. injection

To degrade NETs 1 h after IVH, 300 U (2 µL) of DNase I (04536282001, Roche) was administered via i.c.v. injection at a rate of 0.5 µL/min. The injection was followed by a 2-min needle withdrawal to reduce reflux. Subsequently, 2 µL of DNase I (150 U/μL) was administered via i.c.v. injection every 12 h until the designated time points for each group.

### Neutrophil isolation and identification

Mouse blood samples were obtained via cardiac puncture and collected in anticoagulant tubes. Neutrophils were isolated using gradient centrifugation (P9201, Solarbio). Neutrophil identification was performed by Giemsa staining and IF staining (Ly6g). The isolated neutrophils were then subjected to CFSE staining and NET induction.

### CFSE staining

As previously described 37, the isolated neutrophils were suspended in PBS at a concentration of 5-10×10^6^ cells/mL. CFSE (65-0850-84, Thermo Fisher) was then added to neutrophils in PBS to achieve a final concentration of 1 μM. The mixture was cultured in a dark environment at 37 °C for 20 min. Afterward, CFSE-labeled neutrophils were collected by centrifugation and identified with a 488 nm laser.

### Neutrophil drainage assay

Following anesthesia, the mice were fixed on a stereotactic instrument. To investigate the drainage of neutrophils via meningeal lymphatics, 4 μL of neutrophils labeled with CFSE at a density of 5×10^6^/mL were injected into the lateral ventricle using a microsyringe at a rate of 1 μL/min. Following injection, the device was held in place for 2 min to reduce backflow. Tissue samples were collected 2 h later to examine the drainage of CFSE-labeled neutrophils via mLVs and dCLNs.

### Induction and identification of NETs

Isolated neutrophils were plated at a density of 9×10^6^ cells per well in a 6-well plate. Then, 500 nM PMA (HY-18739, MedChemExpress) was added to each well and incubated in a 37 °C, 5% CO2 incubator for 3 h 40. The samples were collected by scraping the cells and transferred to new centrifuge tubes, which were then centrifuged at 450 g and 4 °C for 10 min. The resulting supernatant contained NETs, whose concentration was measured using the Quant-iT Picogreen dsDNA Assay Kit (P11496, Thermo Fisher). NETs were confirmed by staining with SYTOX Orange (S11368, Thermo Fisher) and visualized using confocal microscopy (LSM 880, Zeiss).

### WB analysis

WB analysis was performed as described previously. For plasma WB analysis, 0.5 μL of plasma was collected 7 days after AAV injection. For WB analysis of the meninges, meningeal tissue homogenate was obtained 24 h after IVH. RIPA lysis buffer and 1% PMSF were used to extract total proteins. The following primary antibodies were added and incubated overnight at 4 °C with gentle agitation: anti-VEGFR3 (goat, 0.1 μg/mL, AF743, R&D), anti-CitH3 (rabbit, 1:2000, ab5103, Abcam), anti-VEGF-C (rabbit, 1:1000, DF7011, Affinity), anti-VE-cadherin (goat, 0.2 μg/mL, AF1002, R&D), anti-PROX1 (rabbit, 1:2000, 11067-2-AP, Proteintech), anti-LYVE-1 (rabbit, 1:1000, 67538S, Cell Signaling Technology), anti-FOXC-2 (rabbit, 1:500, DF3252, Affinity), and anti-CX3CR1 (rabbit, 1:1000, 13885-1-AP, Proteintech). Subsequently, the membrane was incubated at room temperature for 2 h with horseradish peroxidase-conjugated secondary antibodies. The membrane was probed for GAPDH (1:10000, 200306-7E4, ZEN BIO) or ɑ-Tubulin (1:2000, AF5012, Beyotime) as a loading control.

### IF staining

IF staining was performed as previously described. Briefly, 24 h after IVH, the mice were subjected to deep anesthesia and perfused with physiological saline and 4% paraformaldehyde. The meninges and dCLNs were isolated. Then, the meninges and dCLNs were immersed in 4% paraformaldehyde for 24 h and subsequently dehydrated in a 30% sucrose solution for 72 h. Lymph node sections (15 μm) were obtained using a cryostat (Leica, CM1860UV, Germany) and treated with 0.5% Triton for 30 min. Subsequently, the lymph node slices and meninges were blocked with 5% normal goat serum for 2 h and incubated overnight at 4 °C with the following primary antibodies: anti-LYVE-1 (rabbit, 1:200, 67538S, Cell Signaling Technology), anti-LYVE-1 (rat, 1:100, 140-443-82, Thermo Fisher), anti-CX3CR1 (rabbit, 1:100, 13885-1-AP, Proteintech), anti-CitH3 (rabbit, 1:200, ab5103, Abcam), anti-Ly6G (1:200, ab25377, Abcam), anti-VEGFR3 (rabbit, 1:100, ab27278, Abcam), and anti-fibrinogen (sheep, 1:200, PA185429, Thermo Fisher). Next, the samples were incubated with the appropriate secondary antibodies at room temperature for 2 h, followed by staining with DAPI for 5 min.

### LEC culture and identification

Mouse LECs were purchased from Procell Life Science & Technology Co. Ltd. (CP-M023, Pricella) and were cultured and passaged with mouse LEC complete culture medium (CM-M023, Pricella). After three passages, the formal experiment was performed. VEGFR3 staining was used to identify LEC purity.

### Apoptosis assay

Apoptosis was assessed using the ANNEXIN V-FITC/PI cell apoptosis detection kit (BB-4101, Bestbio). LECs in the different experimental groups were trypsinized without EDTA, suspended in binding buffer, and incubated with Annexin V-FITC for 15 min, followed by PI staining for 5 min. Flow cytometry (Canto2, BD, USA) was then performed to analyze the results.

### Cell viability assay

To determine the viability of LECs, a Cell Counting Kit-8 (CCK-8) assay (Beyotime, C0038) was used. LECs were seeded on 96-well plates and treated with varying concentrations of NETs for 24 h. After the addition of 10 µL of CCK-8 solution, the culture medium was incubated at 37 °C for 2 h. The absorbance of each well was measured at 450 nm using a microplate reader (Thermo Scientific MULTISKAN GO).

### TUNEL staining

An Apoptosis Detection Kit (12156792910, Roche) was used to perform TUNEL staining on the meninges and LECs according to the manufacturer's protocol. TUNEL^+^ cells in mLVs were quantified using ImageJ. The results are reported as the number of TUNEL-positive cells per square millimeter (cells/mm^2^) of mLV.

### ELISA

VEGFC (EM0217, Fine test) and FOXC2 (EM6948, Fine test) were analyzed by ELISA. Duplicate measurements were performed using a commercially available ELISA kit to determine the levels of VEGFC and FOXC2 in mouse meninges.

### Quantitative RT‒qPCR

RT‒qPCR was used to quantify the mRNA levels of FOXC2 and VEGFC in the meninges of mice. Twenty-four h after IVH, the meninges were collected, and total RNA was extracted with a commercially available RNA extraction kit according to the manufacturer's instructions. The production of complementary DNA (cDNA) was performed using a reverse transcription kit. Subsequently, real-time quantitative PCR (RT-qPCR) was performed to analyze relative mRNA expression levels using the 2DeltaDeltaCt method. Table S1 provides a list of the primers used in this study. The genotype of the Cx3cr1^-/-^ offspring was validated via PCR using the following primers: F, 5'-GGGTCTCCCTACCTTATTAACTCAA-3'; R1, 5'-GAGAACAGAGGTGTGGTCTCACT-3'; R2, 5'-CACTGAAAGGGTGAACAATTGGAAC-3'. All primers were synthesized by Beijing Tsingke Biotech Co., Ltd.

### NO analysis

To assess the function of lymph flow in the meningeal lymphatic system, NO levels were measured 39. After 24 h of IVH, the meninges were collected, homogenized in PBS buffer containing protease inhibitors, and then subjected to centrifugation to obtain the supernatant. The NO levels in the tissue samples were assessed by using NO assay kits (A012-1-2, Nanjing Jiancheng Biotechnology Institute) according to the instructions provided with the kit.

### Lentiviral infection

The lentiviruses were purchased from GeneChem. Lentiviruses encoding CX3CR1 or a scrambled control were used to transfect LECs. Then, the transfected cells were treated with puromycin for 7 days to select successfully transfected cells, CX3CR1 expression was evaluated, and the cells were then cultured for subsequent in vitro experiments. The knockdown sequences used in this study were Lv-shRNA*cx3cr1-1*: GCAGTTTCACTCACTACACAA, Lv-shRNA*cx3cr1-2*: GCCTGTTATTTGGGCGACATT, and Lv-shRNA*cx3cr1-3*: CCCTTCAGTAACTAGCAGTAT.

### Neurofunctional assessments

#### Open field test

The open field test was performed to measure the overall locomotion behavior of mice 43. The testing instrument was 50 cm × 50 cm × 50 cm in size. The mice were placed in the open field arena and allowed to freely explore without any confinement for 5 min, and their behavior was recorded. Prior to conducting the experiment with the next mouse, we thoroughly eliminated any residual olfactory cues left by the previous mouse. Two independent testers analyzed the data, and ViewPoint behavioral technology software (Lyon, France) was used to measure the distance and average speed of each mouse, which had unrestricted movement.

#### Morris water maze task

Following an established protocol, the water maze task was performed 1 day after IVH 44. In brief, the experimental procedure involved six mice per group, which were subjected to four trials lasting a maximum of 60 s each and were performed over five consecutive training days. Subsequently, on the sixth day, a single 60-s probe trial was administered. The time it took for the mouse to reach the platform during the training days, the frequency of passing through the target area (former platform position), and the duration within the target quadrant during the probe trial were all measured and recorded.

#### Modified neurological severity score (mNSS)

To comprehensively evaluate neurological function, including balance, sensory, and motor functions, we used the mNSS 18. A scale ranging from 0 to 18 was used to grade neurological function, and the scores indicated different levels of injury severity, such as more severe (13-18), moderate (7-12), or mild (1-6) injury.

### Transmission electron microscopy (TEM)

After 24 h of IVH, the mice were anesthetized and subsequently subjected to intracardiac perfusion with a solution of 0.1 mol/L Sorensen's buffer (pH 7.4) containing 4% paraformaldehyde and 2.5% glutaraldehyde. The animals were dissected at 4 °C to retrieve the meninges. The meninges surrounding the transverse sinus (TS) were removed with microscissors, and the TS was sectioned and trimmed into small tissue blocks (1 mm^3^). Subsequently, the samples were fixed with a 1.0% OsO4 solution and dehydrated using a series of ethyl alcohol solutions with increasing concentrations. Once dehydration was complete, the specimens were infused with propylene oxide, embedded in Epon resin, and then sectioned. The stained samples were analyzed with a Philips CM 100 transmission electron microscope (Hillsboro, OR, USA) and digitally documented with a Hamamatsu ORCA-HR camera (Hamamatsu City, Shizuoka, Japan) 45.

### Scanning electron microscopy (SEM)

After 24 h of IVH, the animals were euthanized under deep anesthesia, and the meninges were promptly dissected. Following the established procedure, meninges samples containing the specified volume (1 mm^3^) in TS were processed for SEM (FEI Quanta 450; FEI Company, Hillsboro, Oregon, USA) as previously described 46. In brief, the tissues were fixed in Sorensen's phosphate-buffered glutaraldehyde (4%, pH 7.4) for 48 h. Following fixation in 1% osmium tetroxide, the samples were subjected to ethanol gradients to remove water and dried. Subsequently, the mounted samples were sputter coated with platinum and prepared for examination.

### Live animal imaging

To prevent the attenuation of fluorescent signals, the anterior neck skin of the mouse was shaved. A volume of 5 μL of OVA647 (2 mg mL^-1^ in PBS, O34784, Thermo Fisher) was intracerebrally injected into the mice at a rate of 1 μL/min. The fluorescence intensity was measured 2 h after injection of OVA647 using an In Vivo Imaging System (IVIS) Spectrum (Perkin-Elmer). The obtained results were analyzed using Living Image 4.7.3 software (Perkin-Elmer).

### MRI and ventricular volume analysis

The mice were administered a 2% isoflurane/air mixture to ensure anesthesia during the entire MRI procedure. Using a 7.0-T Varian MR scanner (Bruker, USA), the MRI scans were carried out with a *T*_2_* gradient-echo sequence and a *T*_2_ fast spin‒echo sequence. In this study, the imaging parameters used included a view field measuring 30 mm × 30 mm and 24 coronal slices with a thickness of 0.6 mm. The ventricular systems were subsequently reconstructed in 3D using 3D Slicer software 17.

### Quantitative RNA sequencing

For tissue samples, the mice were euthanized after 24 h of IVH. For cell samples, LECs were harvested after being cocultured for 24 h. RNA-Seq was performed according to the manufacturer's protocol, and the results were analyzed by Wuhan Seqhealth Co. Briefly, TRIzol reagent was used to extract total RNA from ventricular wall tissue and LECs. Following quantification and purification, total RNA was subjected to reverse transcription to generate cDNA, which was subsequently used to synthesize U-labeled second-stranded DNA. After ligation, the products were subjected to PCR amplification, resulting in the generation of a cDNA library with an average insert size of 300 bp (50 bp). The expression levels of all transcripts were quantified by determining the fragments per kilobase per million reads.

### Bioinformatic analysis

To examine the biological pathways in ventricular wall tissue, the Gene Orthology (GO) and Kyoto Encyclopedia of Genes and Genomes (KEGG) databases were used. To examine the active biological pathways in ventricular wall tissue, gene set enrichment analysis (GSEA) and gene set variation analysis (GSVA) were performed. To assess the presence of M1 and M2 macrophage-related genes in the samples, we used single-sample gene set enrichment analysis (ssGSEA). Using the GSVA package in R and setting the method parameter to ssGSVA, we computed ssGSEA scores for each sample, which yielded normalized enrichment scores. These scores indicate the extent to which genes associated with M1 and M2 macrophages are coordinately up- or downregulated within each sample with a multiple comparisons test. To investigate the active biological pathways in LECs, Gene Ontology (GO) analysis of the differentially expressed genes was conducted using KOBAS software (version: 2.1.1). Differentially expressed genes were identified with thresholds of P <0.05 and |log2(fold change)| ≥ 1.

### Statistical analysis

GraphPad Prism was used for data analysis and visualization, and the data are presented as the means ± SEMs. Sample sizes were determined using a previously determined sample size calculator, which assumed a significance level (α) of 0.05, a two-tailed test, and a desired power of 80%. The findings indicated that a minimum of six mice per group was needed. Mice that unexpectedly died during the experimental process were excluded from the study. For data that showed a normal distribution and homogeneity of variance, t-tests or one-way ANOVA was employed, while non-parametric tests were used for data that did not show a normal distribution or homogeneity of variance, as well as for most score data. Data from immunostaining, WB analysis, MRI, and behavioral experiments were analyzed using one-way ANOVA. For single comparisons, we used Student's t test, and for multiple comparisons, analysis of variance with post hoc Bonferroni-Dunn correction was used, depending on the specific experiment. A significance level of p < 0.05 was used to determine statistical significance (ns: P > 0.05, *P < 0.05, **P < 0.01, ***P < 0.001, and ****P < 0.0001).

## Results

### mLVs are involved in the development of hydrocephalus after IVH

**Figure 2:**
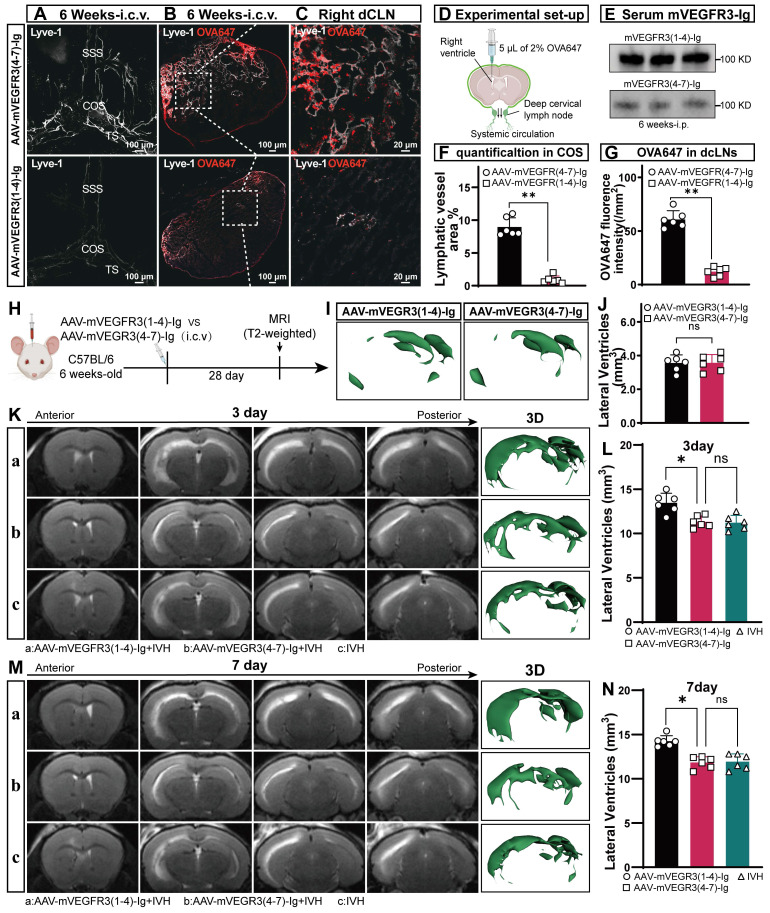
** mLV impairment exacerbates post-IVH hydrocephalus.** Mice were given an i.c.v. injection of AAV-mVEGFR3(1-4)-Ig or AAV-mVEGFR3(4-7)-Ig. After day 28, the mice developed IVH. **(A)** Following the i.c.v. injection of specific AAVs into six-week-old mice, representative photomicrographs of LYVE1-stained mLVs around the COS were obtained four weeks later. **(B-C)** Following the schematic functional assay described in **(D)**, representative photomicrographs of LYVE1-stained dCLNs (gray) with OVA647 (red) were obtained. **(E)** WB analysis revealed mVEGFR3-Ig protein levels in serum one week after i.p. AAV injection. **(F)** Quantification of mLVs in the experiments shown in **(A)** (n = 6, Student's t test). **(G)** Measurement of the fluorescence intensity of OVA647 within dCLNs (n = 6, Student's t test). **(H)** T2-weighted MRI was used to evaluate alterations in the volume of the lateral ventricles four weeks after i.c.v. injection of the specified AAVs into six-week-old mice. **(I)** Representative images of the 3D reconstruction of the brain ventricles in mice in both groups. **(J)** Quantification of the volume of the lateral ventricles in the two groups (n = 6, Student's t test). **(K, M)** This illustration shows representative T2-weighted images and 3D reconstructions of the lateral ventricles on days 3 and 7 following IVH in the AAV-mVEGFR3(1-4)-Ig+IVH group, AAV-mVEGFR3(4-7)-Ig+IVH group, and IVH group. **(L, N)** Quantification of the volume of the lateral ventricles in each group (n = 6, one-way ANOVA). The results are presented as the mean ± SEM. ns: P > 0.05, *P ≤ 0.05, **P ≤ 0.01.

AAV-mediated knockdown of VEGFR3 via i.c.v. injection is an effective method for ablating lymphatic vasculature 32, 33. To assess the AAV transduction efficiency, mice were administered AAV-mVEGFR3(4-7)-Ig-GFP via i.c.v. Immunofluorescence staining for LYVE-1 was conducted four weeks post-virus injection, revealing stable expression of AAV-mVEGFR3(4-7)-Ig-GFP in LECs (Figure S1). For mice receiving AAV via i.p. injection, the AAV transduction efficiency was evaluated via WB analysis of plasma on day 7 post-injection, which demonstrated sustained expression of mVEGFR3-Ig in the serum (Figure 2E). To determine whether AAV-mVEGFR3(1-4)-Ig injection ablates mLVs, the mice received i.c.m. injection of OVA647 four weeks after AAV i.c.v. injection (Figure 2D). We examined the impact on mLVs by performing immunostaining with LYVE-1 antibodies to identify the ablation of mLVs (Figure 2A). A reduction in the number of LYVE-1^+^ cells in the COS of the meninges was observed in AAV-mVEGFR3(1-4)-Ig group mice after i.c.v. injection (Figure 2F). Mice injected with AAV were subjected to i.c.m. injection of OVA647, and the subsequent distribution of OVA647 in the dCLNs was assessed 2 h after injection (Figure 2B, C). Two h after injection, OVA647 was observed in the dCLNs of control mice, whereas mice with ablation exhibited minimal levels of OVA647 (Figure 2G). These findings suggest that the lymphatic drainage capacity of mLVs was compromised in mice infected with AAV-mVEGFR3(1-4)-Ig.

To further investigate the involvement of mLVs in the development of hydrocephalus after IVH, we induced an autologous blood IVH model in mice that did or did not have preexisting meningeal lymphatic dysfunction. We first evaluated T2-weighted imaging in the control and ablation groups 4 weeks after viral injection and found that ablating mLVs did not affect the lateral ventricular volume in normal mice (Figure 2H, I, J). Next, we used T2-weighted imaging scans and 3D reconstruction images to quantify the lateral ventricular volumes three and seven days after IVH to assess the extent of hydrocephalus in each group (Figure 2K, M). Our findings indicated that mice with preexisting meningeal lymphatic dysfunction in the AAV-mVEGFR3(1-4)-Ig+IVH group exhibited exacerbated hydrocephalus, as evidenced by the T2 images obtained at 3 and 7 days (Figure 2L, N). These observations demonstrate that mLV drainage is involved in the development of hydrocephalus after IVH.

### Prior lymphatic ablation aggravates IVH-induced inflammation

**Figure 3:**
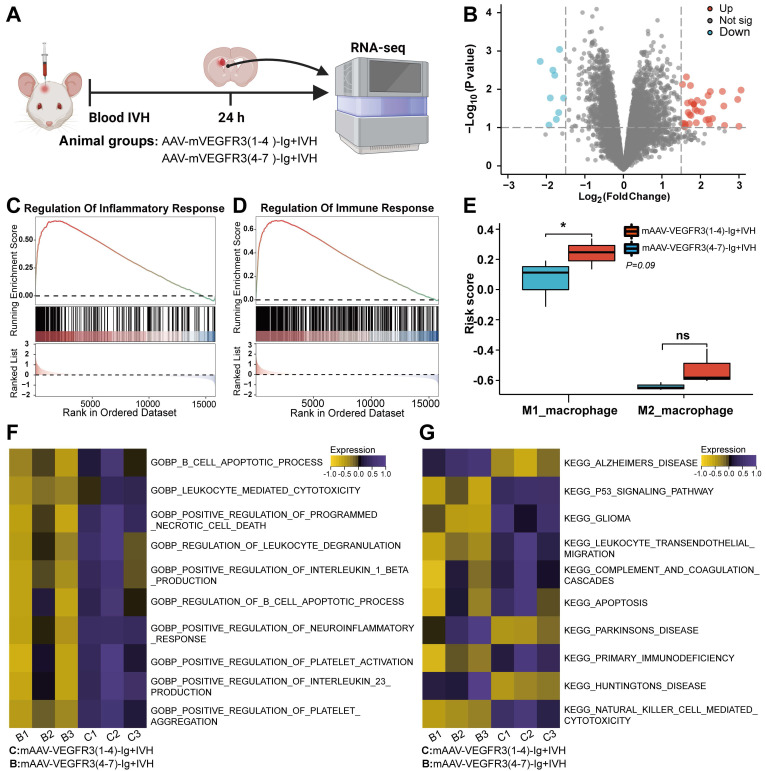
** The presence of preexisting meningeal lymphatic dysfunction leads to changes in gene expression 24 h after IVH.** The mice were administered an i.c.v. injection of AAV-mVEGFR3(1-4)-Ig or AAV-mVEGFR3(4-7)-Ig. Following a 28-day period, the mice were subjected to IVH. Twenty-four h after injury, RNA was isolated from the ventricular wall. Two experimental groups containing three samples each were subjected to bulk RNA sequencing. **(A)** Schematic of the experimental design. **(B)** Volcano plots show the number of significantly differentially expressed genes in each group. **(C-D)** GSEA showing the significant upregulation of factors associated with the regulation of the inflammatory response and regulation of immune response pathways in tissues in the AAV-mVEGFR3(1-4)-Ig+IVH group compared to those in the AAV-mVEGFR3(4-7)-Ig+IVH group. **(E)** ssGSEA showing significantly increased M1 microglial activation in the AAV-mVEGFR3(1-4)-Ig+IVH group compared to the AAV-mVEGFR3(4-7)-Ig+IVH group (n = 3, Student's t test). **(F)** GO_GSVA showing pronounced activation of the neuroinflammatory response, programmed necrotic cell death, and platelet activation pathways in the AAV-mVEGFR3(1-4)-Ig+IVH group. **(G)** KEGG_GSVA showing significant upregulation of pathways involved in immunodeficiency, apoptosis, and thrombosis in the AAV-mVEGFR3(1-4)-Ig+IVH group. The data are means ± SEMs (SD), ns: P > 0.05, *P ≤ 0.05.

To investigate the specific effects of prior meningeal lymphatic ablation on brain injury, we administered AAV-mVEGFR3(1-4)-Ig by used i.c.v. injection to ablate mLVs prior to the induction of IVH. Then, we performed bulk RNA sequencing on the lateral ventricular walls of mice 24 h after IVH and compared those with ablated mLVs to those without (Figure 3A). Differential analysis identified a total of 258 differentially expressed genes with a LogFC ≥ 1 in the group with mLV ablation (Figure 3B). Interestingly, GSEA revealed a significant upregulation of genes related to inflammatory regulation and immune response regulation in the group with preexisting lymphatic dysfunction before IVH, and the effect occurred as early as 24 h postinjury (Figure 3C, D). Gene Ontology (GO) and GSVA revealed a significant increase in pathways related to leukocyte-mediated cytotoxicity, the regulation of leukocyte degranulation, positive regulation of interleukin-1β production, platelet aggregation, positive regulation of the neuroinflammatory response, positive regulation of interleukin 23 production, and positive regulation of programmed cell death in the disrupted group compared to the Control group (Figure 3F). Next, we examined the changes in M1 and M2 macrophage-related genes in the samples. We used ssGSEA, which is an extension of conventional GSEA, to calculate individual enrichment scores for each combination of a sample and gene set. Our findings indicated that individuals with preexisting ablation of meningeal lymphatic function before IVH exhibited increased expression of genes associated with M1 macrophage-mediated inflammation 24 h postinjury (Figure 3E). In summary, our results suggest that individuals with preexisting impairment in meningeal lymphatic function prior to IVH exhibit increased expression of genes involved in leukocyte-mediated and M1 macrophage-mediated inflammation 24 h after injury. Additionally, our results demonstrate that preexisting meningeal lymphatic dysfunction prior to head injury has an adverse effect on the expression of genes associated with neuronal health.

We next examined whether mice with preexisting lymphatic ablation (Ablated + IVH) exhibited overlapping gene signatures linked to neurodegeneration. We used the KEGG database and GSVA and showed marked upregulation of pathways associated with neurodegenerative diseases, including Alzheimer's, Huntington's, and Parkinson's diseases (Figure 3G). This finding suggests that mice with lymphatic ablation prior to IVH share signatures with various neurological disease states.

### Neutrophils, NETs and fibrin are present in the meningeal lymphatic system after IVH

**Figure 4:**
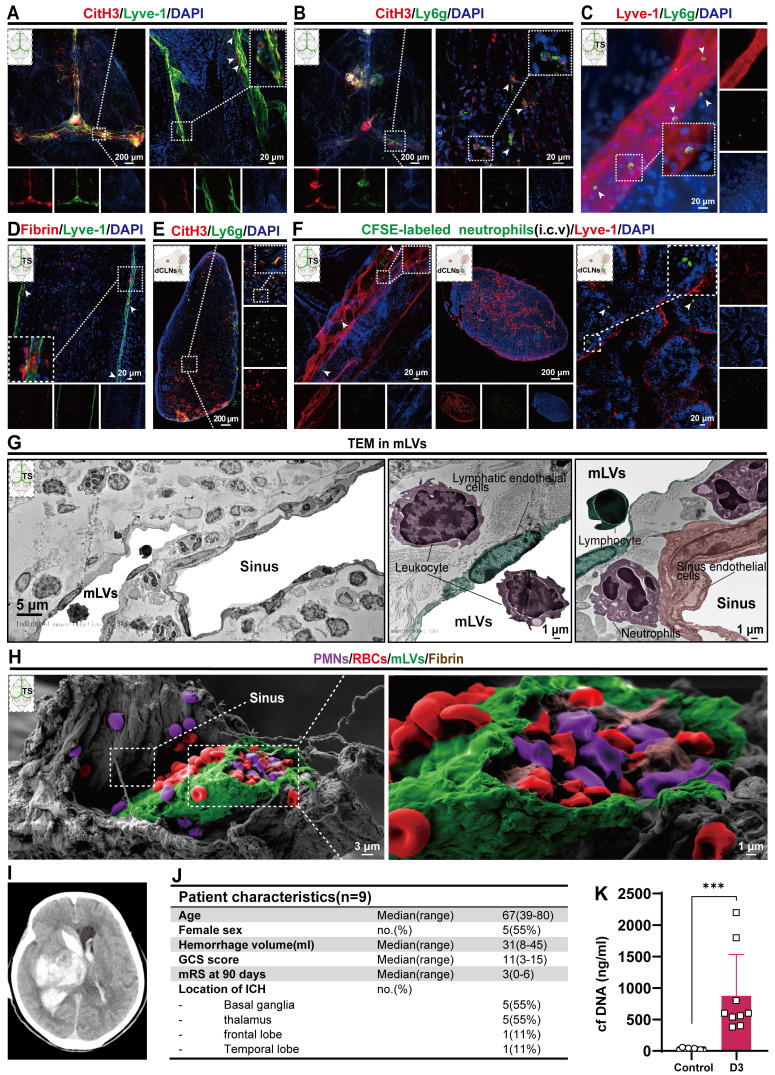
** Neutrophils, NETs, and fibrin were present in mLVs after IVH.** Twenty-four h after IVH, the meninges and CLNs of mice were collected for observation.** (A)** Representative fluorescence photomicrographs showing the immunolabeling of CitH3 in mLVs following IVH. **(B)** Representative images showing NETs in the TS. **(C)** Representative fluorescence photomicrographs showing the immunolabeling of Ly6g in mLVs after IVH. **(D)** Representative fluorescence images showing the immunolabeling of fibrin in mLVs after IVH. **(E)** Representative fluorescence images showing the immunolabeling of NETs in dCLNs after IVH. **(F)** Images showing CFSE-labeled neutrophils in Lyve1^+^ mLVs and dCLNs. **(G)** TEM revealed the ultrastructural composition of the TS, including sinus ECs, LECs, lymphocytes, neutrophils, and leukocyte interactions after 24 h of IVH. **(H)** SEM revealed the ultrastructural composition of the TS, including the sinus, mLVs, and entrainment of erythrocytes, polymorphonuclear neutrophils, and fibrin within mLVs following IVH. **(I)** Representative cases of ICH-IVH. CT imaging showing the presence of hyperdense blood accumulation in the right basal ganglia and ventricular system. **(J)** Overview of patient characteristics. **(K)** Assessment of double-stranded DNA (dsDNA) concentrations in CSF within a three-day timeframe in both control individuals and patients diagnosed with ICH-IVH (n =9, non-parametric tests). The data are means ± SEMs (SD), *** p < 0.001.

In our previous study, we observed a significant neutrophil infiltration and the formation of NETs following ICH/IVH, which hindered the fibrinolytic effect of tPA on the hematoma 38. Our previous research demonstrated that NETs induced by SAH contribute to early brain injury via fibrin formation 29. Here, we examined whether NETs were present in the mLVs of IVH and investigated the interaction between neutrophils, NETs, fibrin, and the meningeal lymphatic system. To determine whether NETs were present in the meningeal lymphatic system, mice were euthanized 24 h after IVH. We examined changes in dCLNs and mLVs by immunostaining with antibodies targeting CitH3, LYVE-1, Ly6g, or fibrin to detect their interaction with the meningeal lymphatic system. Our findings revealed that CitH3 was present within LYVE-1**-**positive mLVs (Figure 4A). Concurrently, the results revealed a significant number of neutrophils and CitH3^+^ neutrophils in the TS of IVH mice (Figure 4B). We also observed a significant number of Ly6g^+^ neutrophils in the mLVs of mice with IVH (Figure 4C). Recent studies have reported the involvement of NETs in inducing fibrin deposition in multiple diseases, such as COVID-19 and SAH 29, 30. Consequently, we next examined whether fibrin deposition occurred in mLVs following IVH. Our results demonstrated the presence of fibrin within LYVE-1^+^ mLVs (Figure 4D). Finally, we found that NETs were present in dCLNs after 24 h of IVH (Figure 4E). Overall, our findings suggest that neutrophils, NETs, and fibrin were widely present in the meningeal lymphatic system after IVH.

mLVs can effectively drain senescent astrocytes and brain metabolic waste in AD and can facilitate the drainage of erythrocytes after SAH 21, 22, 47. Therefore, we examined whether neutrophils from the peripheral blood could be drained through the meningeal lymphatic system, given that they are the earliest peripheral immune cells to arrive following IVH. CFSE-labeled neutrophils with complete cell morphology were observed within LYVE-1^+^ mLVs and dCLNs (Figure 4F).

To examine the detailed morphology of the mLV, we performed TEM to examine coronal sections of the TS after 24 h of IVH. TEM revealed that the dural meninges consisted of a dense collagen network (Figure 4 G-left). The dural sinuses were closely associated with the lymphatic endothelial vasculature, which exhibited discontinuous junctions, thus verifying the permissive nature of lymphatic vessels and dural blood for the exchange of fluid and cells (Figure 4G-right). Consistent with IF staining of mLVs, we observed the presence of leukocytes (purple) and lymphocytes (green) in mLVs at 24 h after IVH (Figure 4G-right). Additionally, sinus-associated leukocyte populations with macrophage characteristics (purple) were observed around the sinus and mLVs (Figure 4G).

To further investigate the morphological characteristics of mLVs, we used SEM to analyze coronal sections through the TS after 24 h of IVH. We confirmed that mLVs were adjacent to the sinuses, and a significant number of RBCs were observed (Figure 4H-left). Importantly, we discovered the deposition of RBCs (red), leukocytes (purple), and fibrin (brown) within mLVs after IVH (Figure 4H-right). These results were consistent with the IF staining results.

Next, we examined whether NETs were present in CSF after ICH/IVH. To examine this, CSF samples were collected within three days from ICH/IVH patients (Figure 4J). NETs were quantified by measuring dsDNA using SYTOX Green. Figure 4J and table S2 show the baseline characteristics of IVH patients included in this study. Figure 4I shows imaging from one representative ICH/IVH patient: the CT scan of a 76-year-old female patient shows right basal ganglia hemorrhage extending into the ventricle, followed by hematoma evacuation and lateral ventricle drainage surgery. Patients in the Control group underwent lumbar puncture to exclude meningitis or brain hemorrhage, and no intracranial abnormalities were detected. Intriguingly, the CSF of ICH/IVH patients exhibited a significant increase in cfDNA levels compared to those in the Control group (Figure 4K).

### Degrading NETs improves hydrocephalus and neurologic dysfunction after IVH

**Figure 5:**
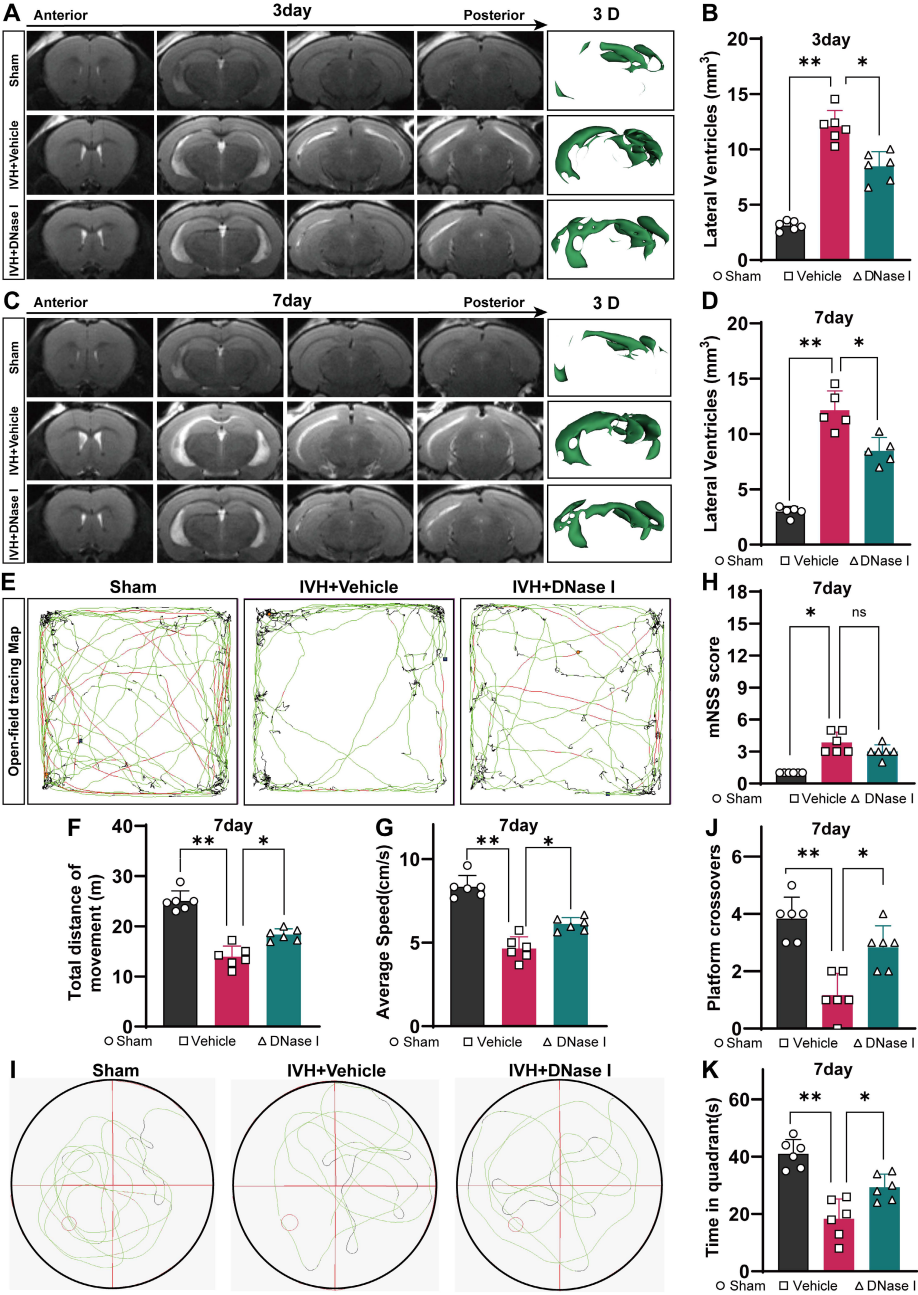
** Hydrocephalus and neurologic deficit evaluation index after IVH.** C57BL/6 mice were i.c.v. injected with or without DNase I after IVH. **(A, C)** Illustrative T2-weighted images and three-dimensional reconstructions of the lateral ventricles were acquired three and seven days later in the Sham, IVH, and IVH+DNase I groups. **(B, D)** Measurement of lateral ventricle volumes using associated T2-weighted images (n = 6, 5, one-way ANOVA). **(E)** Illustrative movement trajectories of mice in the different experimental groups 7 days post-IVH. **(F)** Summarized data showing the distance traveled in the open field during the experiment (E) (n = 6, one-way ANOVA). **(G)** Average speed of the different groups in the open field test (n = 6, one-way ANOVA). **(H)** mNSSs of the different groups (n = 6, non-parametric tests). **(I)** Illustrative movement trajectories and recognition memory performance in each group 7 days post-IVH.** (J)** Comparative analysis of the number of crossings in the previous target area in the different groups (n = 6, one-way ANOVA).** (K)** Assessment of the time spent in the target quadrant during the probe trial (n = 6, one-way ANOVA). The data are means ± SEMs (SD), *P < 0.05, **P < 0.01.

NET degradation has been shown to improve CSF circulation in pneumococcal meningitis 28. Here, we examined whether NET degradation could improve hydrocephalus after IVH. Mice were treated with DNase I via i.c.v. injection after IVH, and then we assessed the degree of hydrocephalus in control, injured, and DNase I-treated mice by quantifying the volume of the lateral ventricle using T2 MRI scans and 3D reconstruction images three and seven days after IVH (Figure 5A, C). We revealed that IVH induced more pronounced lateral ventricular dilation than DNase I-treated IVH (DNase I+IVH). Severe hydrocephalus was evident in both groups three days posthemorrhage and remained persistent until day seven (Figure 5 B, D). In comparison to the Sham group, the IVH groups exhibited significantly increased lateral ventricular volumes, suggesting the presence of hydrocephalus.

Multiple behavioral tests were performed to assess the overall behavioral performance of mice in the groups. As previously reported, motor function was assessed using the open field test on day 7 after IVH 43 (Figure 5E). On day 7 after IVH, mice in the IVH group exhibited notable deficits in motor function, including decreases in total movement and average speed, in comparison to mice in the Sham group (Figure 5F, G). Notably, mice with IVH exhibited improved locomotion recovery following intraventricular injection of 300 U/2 μL DNase I, whereas treatment with saline had no discernible effect on functional recovery.

The mNSS was used to comprehensively evaluate neurofunction and revealed severe neurological deficits in the hemorrhage groups. Overall, neurological deficits were not alleviated in the DNase I + IVH group compared with the IVH group (Figure 5H). Furthermore, cognitive function was assessed using the water maze test, which is a classic method for evaluating cognitive function (Figure 5I). During the probe trial to evaluate spatial memory, when the hidden platform was removed, the administration of DNase I resulted in a significant increase in the number of former platform crossings by IVH mice (Figure 5J). Additionally, the target quadrant time was significantly higher in the DNase I-treated group than in the IVH group (Figure 5K). Taken together, these results suggest that the IVH groups exhibited significant neurological deficits and hydrocephalus in the acute phase, and NET degradation contributed to the amelioration of hydrocephalus and improvements in neurological deficits during this phase.

### NET degradation improves the ability of the mLV to remove biological molecules after IVH

**Figure 6:**
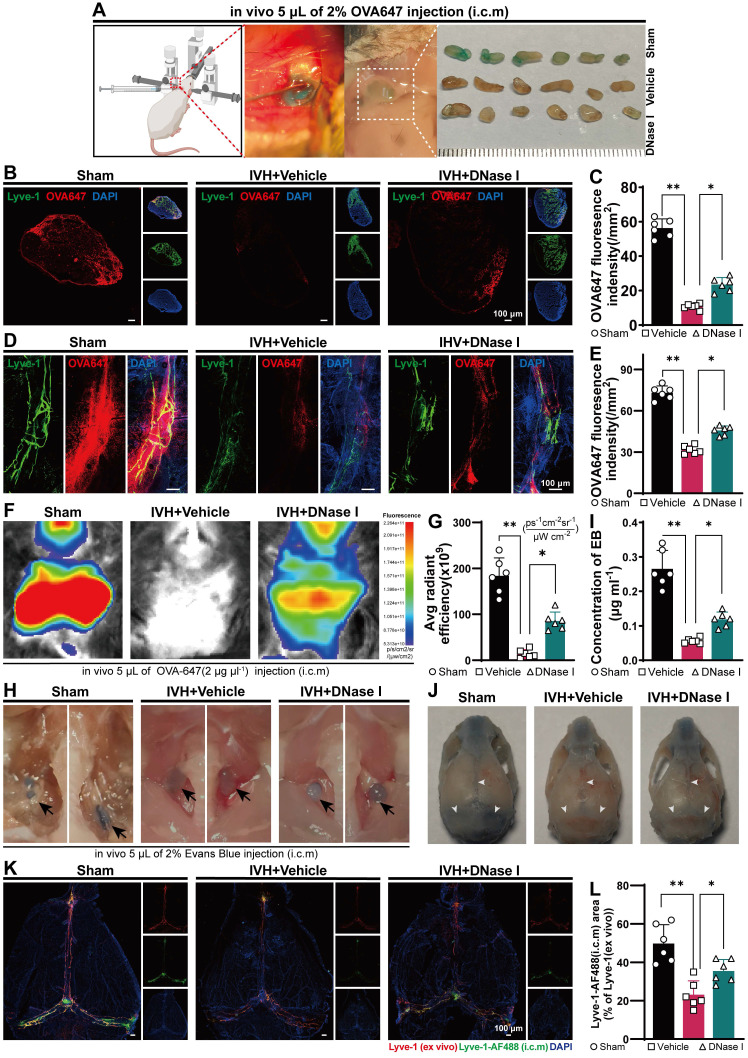
** NET degradation improves the ability of mLVs to remove biological molecules after IVH.** Twenty-four h after IVH, C57BL/6 mice were i.c.m. injected with OVA647, EB, or LYVE-1-AF^488^ antibodies. **(A)** Representative images of right sCLNs were taken in Sham, DNase I-treated IVH, and IVH mice 2 h after i.c.m. injection of OVA647. **(B)** Representative fluorescence photomicrographs showing LYVE-1 staining and OVA647 in dCLNs 2 h after i.c.m. injection of OVA647. **(C)** Evaluation of the average intensity of OVA647 fluorescence in dCLNs on the right side (n = 6, one-way ANOVA). **(D)** Representative images of mLVs in the right TS showing the drainage of fluorescent OVA647 in the different groups.** (E)** Measurement of the mean fluorescence intensity of OVA647 in mLVs in the right TS (n = 6, one-way ANOVA). **(F)** Images of live mice in the ventral position were captured 2 h after the injection of OVA647 by using an in vivo animal imaging system. **(G)** The fluorescence intensity of OVA647 in the cervical region was quantified (n = 6, one-way ANOVA). **(H)** Images showing representative dCLNs in Sham, IVH, and DNase I-treated mice were obtained 2 h after i.c.m. injection of 2% EB. dCLNs are highlighted by the blocked arrows. **(I)** EB concentrations in dCLNs (n = 6, one-way ANOVA). **(J)** The distribution of EB in mLVs was assessed in the Sham, IVH and DNase I-treated groups. White narrows: EB. **(K)** Representative images show meninges stained with anti-LYVE-1 (ex vivo) and AF^488^-conjugated anti-LYVE-1 (i.c.m.). **(L)** Quantification of the the area fraction (%) of meningeal lymphatics labeled with AF^488^-conjugated LYVE-1 antibodies (i.c.m.), which was then divided by the total area of meningeal lymphatics (n = 6, one-way ANOVA). The data are means ± SEMs, *P < 0.05, **P < 0.01.

Because NET degradation improved hydrocephalus after IVH, we next examined whether NET degradation improved meningeal lymphatic drainage. The biological molecule OVA647 was i.c.m. injected into mice with or without DNase I treatment after IVH (Figure 6A). The presence of OVA647 in mLVs of TS was examined over a 30-min period (Figure 6D), while the presence of OVA647 in draining dCLNs was examined over a 2-h period (Figure 6B). Interestingly, DNase I treatment restored OVA647 deposition in the mLVs of mice with IVH (Figure 6E). Two h after injection, OVA647 was detected in the Control and DNase I-treated groups but was hardly detected in the vehicle group (Figure 6C). Overall, DNase I treatment of mice with IVH promoted OVA647 drainage in dCLNs, whereas saline treatment produced no effect on drainage in IVH mice.

To further investigate the efficacy of mLVs in removing biological molecules, we used in vivo real-time fluorescence imaging to monitor the changes in the biodistribution of OVA647 after intracerebral microinjection (i.c.m.) in IVH mice. Images were taken from the ventral perspective 2 h after administration (Figure 6F). Following i.c.m. administration of OVA647, fluorescent signals were observed in the sCLNs of control mice, but these signals were weak in IVH model mice. However, mice treated with DNase I exhibited decreases in the fluorescence intensity compared to control mice but significantly higher fluorescence intensity than IVH mice (Figure 6G).

Next, we explored the impact of DNase I treatment on CSF flow through lymphatic drainage. EB was intracranially injected into the cisterna magna. This choice was based on EB's high affinity for serum albumin, which facilitates its preferential elimination through the lymphatic system. Then, the mice were treated with 300 U DNase I via i.c.v. administration, and the Control group was given saline. Thirty min later, the presence of EB in the meninges, specifically in the superior sagittal sinus (SSS), TS, and COS, was widely observed in the Sham group, as shown in Figure 6J. Conversely, EB was not detected in the IVH group. Importantly, mice treated with DNase I exhibited more EB than untreated IVH mice (Figure 6J). Two h after the injection of EB, an increase in EB was observed in the dCLNs of control and DNase I-treated mice, while only a minor amount of EB was observed in IVH mice (Figure 6H). Moreover, we found that EB concentrations in dCLNs were higher in DNase I-treated mice than in untreated mice (Figure 6I). These results demonstrate that the ability of mLVs to effectively transport constituents into dCLNs was compromised in IVH mice, whereas NET degradation could increase the flow of CSF.

To evaluate whether DNase I treatment could increase the rate of meningeal lymphatic flow after IVH, we administered AF^488^-conjugated anti-LYVE-1 or saline into the cisterna magna on day 1 after IVH induction. Thiry min later, the meninges were collected and stained for LYVE-1 using an AF^555^-conjugated secondary antibody. Examination of the fraction of mLVs labeled with AF^488^-anti-LYVE-1 antibody following intracisternal magna administration offered insight into the rate of meningeal lymphatic flow (Figure 6K). A markedly higher percentage of meningeal lymphatics labeled by AF^488^-anti-LYVE-1 (i.c.m.) was observed in the DNase I-treated group than in the vehicle group (Figure 6L), and the labeled lymphatics were located on the TS. Collectively, these findings indicate that the groups with IVH exhibited impaired mLV drainage during the acute phase, and NET degradation contributed to the amelioration of mLV drainage during this phase.

### NET degradation improves mLV damage

**Figure 7:**
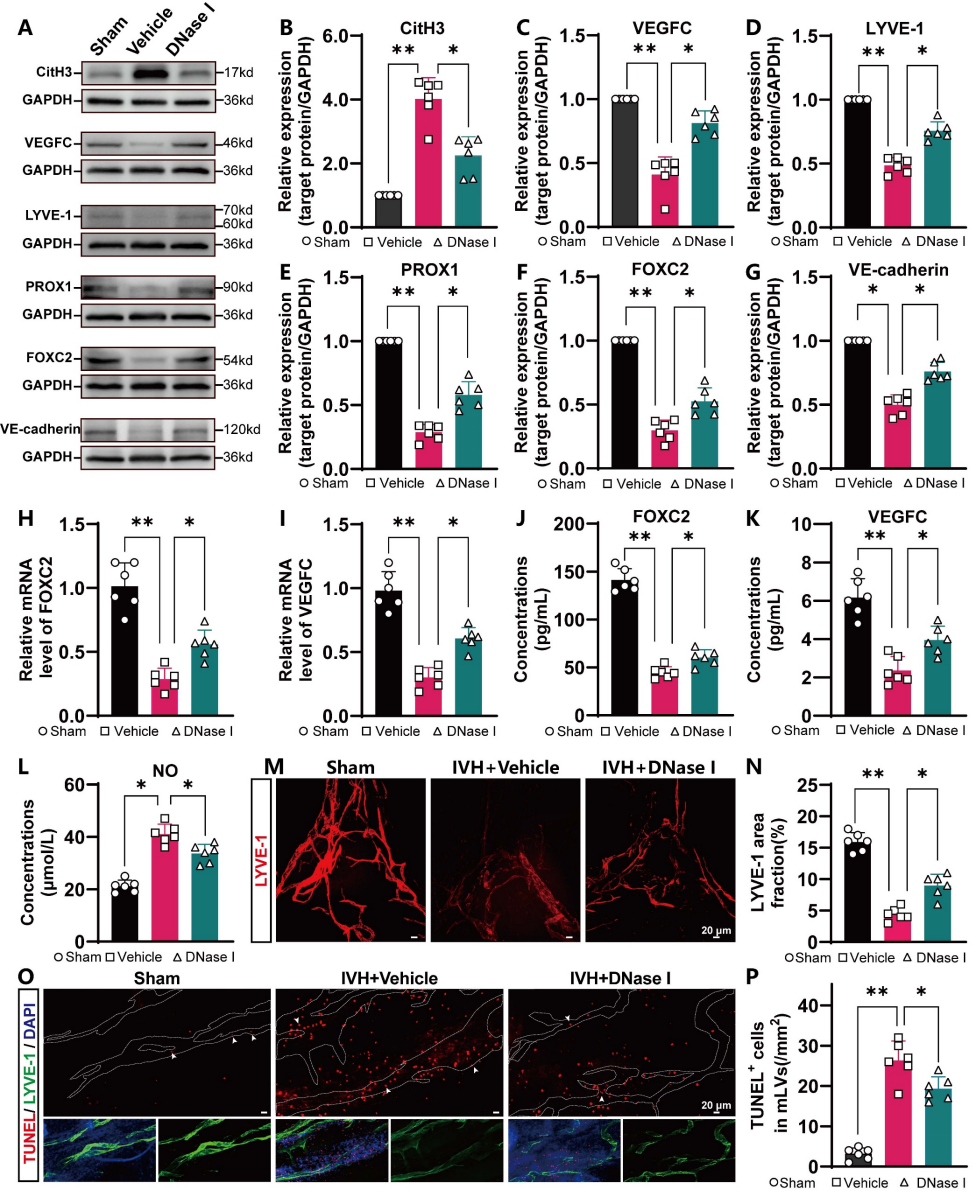
** NET degradation improves mLV damage.** C57BL/6 mice were i.c.v. injected with or without DNase I after IVH. Twenty-four h post-IVH, the meninges were collected for observation. **(A)** Representative WB images showing the expression of CitH3, VEGFC, LYVE-1, PROX1, FOXC2, and VE-cadherin in the meninges in the Sham, IVH, and IVH+DNase I groups 24 h after IVH. GAPDH was used as an internal reference protein. **(B-G)** Quantification of CitH3, VEGFC, LYVE-1, PROX1, FOXC2, and VE-cadherin expression (n = 6, one-way ANOVA). **(H, I)** Relative mRNA levels of FOXC2 and VEGFC in the meninges in the various experimental groups (n = 6).** (J, K)** The concentrations of VEGFC and FOXC2 in the meninges were quantified by ELISA (n = 6, one-way ANOVA). **(L)** NO concentrations in the meninges were quantifid in the different groups 24 h after IVH (n = 6, one-way ANOVA). **(M)** A representative image showing the IF staining of the meninges with anti-LYVE-1 antibodies (red) in the COS.** (N)** The extent of anti-LYVE-1 antibody staining in the meninges of the COS was quantified and is presented as a percentage of the total area (n = 6, one-way ANOVA). **(O)** Images showing TUNEL and LYVE-1 staining in the TS of Sham-, IVH-, or DNase I-treated mice were selected as representative examples 24 h after IVH. **(P)** The number of TUNEL-positive LECs surrounding the TS in the meninges of mice (n = 6, one-way ANOVA). *P < 0.05, **p < 0.01. Data are means ± SEMs.

Because NET degradation improved mLV drainage after IVH, we next examined whether NET degradation improved meningeal lymphatic function and damage. To evaluate the level of CitH3 in the meninges, the meninges were harvested and subjected to WB analysis. The results indicated a significant increase in the levels of CitH3 in the meninges in the IVH group compared to the Sham group (Figure 7A). However, treatment with DNAse I reduced CitH3 levels (Figure 7B). Studies have revealed that the expression of FOXC2, VEGFC, PROX1, LYVE-1, and VE-cadherin plays an essential role in lymphangiogenesis and the subsequent maturation of mLVs 32, 48, 49. VEGFC stimulates the production of LECs, which migrate and contribute to the formation of primary lymphatic vessels. As a pivotal marker, FOXC2 is instrumental in the maturation of primary lymphatic vessels, facilitating their normal development and optimal functioning. Furthermore, evidence suggests that a decrease in FOXC2 expression contributes to the development of lymphedema 50. The level of LYVE-1, a marker exclusive to LECs, is directly associated with the number of LECs. The presence of the transcription factor PROX1 is crucial for the proper function and preservation of lymphatic valves. The involvement of VE-cadherin in mLVs has been shown to be organ specific, and it contributes to the establishment and preservation of distinct lymphatic beds, such as dermal lymphatics, lacteals, and mesentery, as well as in the developmental processes of intraluminal lymphatic valves. The WB results indicated increased expression of lymphangiogenesis markers, including PROX1, FOXC2, VEGFC, VE-cadherin and LYVE-1, in the meninges in the DNase I-treated group compared with IVH group at 24 h (Figure 7C-G). The RT‒qPCR and ELISA results demonstrated that after DNase I treatment, the mRNA and protein expression of FOXC2 and VEGFC in the meninges was increased (Figure 7H-K). The lymphatic area in the COS was quantified to measure changes, as shown by LYVE-1 staining (Figure 7M). Our results demonstrated a significant reduction in the LYVE-1 staining area in the IVH group compared to the Sham group (Figure 7N). However, the DNase I-treated group exhibited a larger LYVE-1 staining area than the IVH group. Therefore, our results suggested that DNase I treatment could improve lymphangiogenesis and the maturation of mLVs after IVH.

Lymphatic contraction plays a pivotal role in the dynamic process of lymphatic circulation and is essential for maintaining the homeostasis of the circulatory system. NO is a crucial modulator of lymphatic pumping and is indispensable for the regulation of lymphatic function. Under physiological conditions, rhythmic fluctuations in the bioactive molecule NO are involved in controlling lymphatic vessel contraction, tension, and relaxation 51. Consequently, NO levels in the meninges were measured following IVH with or without DNase I treatment. The DNase I-treated group exhibited a notable reduction in the concentration of NO in the meninges compared to the IVH group (Figure 7L), suggesting that DNase I treatment could improve mLV contractility and lymphatic circulation.

To assess the extent of damage in mLVs following IVH, the meninges were harvested and subjected to TUNEL staining and LYVE-1 staining (Figure 7O). The extent of damage was assessed by quantifying the number of TUNEL-positive cells in mLVs. We found a significant reduction in TUNEL-positive cells in mLVs in the DNase I-treated group compared to the IVH group (Figure 7P). Thus, DNase I treatment may improve meningeal lymphatic endothelial activity by improving the morphology, production, damage, and function of meningeal lymphatic endotheliocytes after IVH, suggesting that DNase I treatment may promote the recovery of LEC injury following IVH.

### NETs induce LEC damage *in vitro*

**Figure 8:**
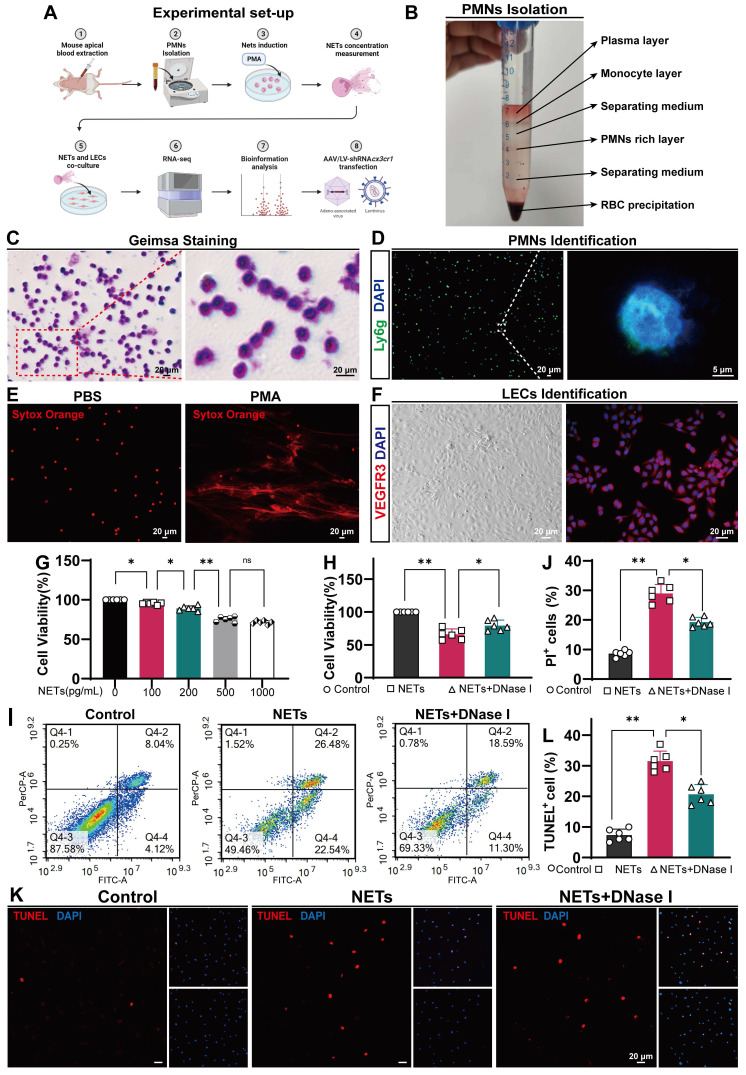
** NETs induce LEC damage.** NET formation was induced by PMA, and the purified NETs, as well as PBS- and DNase I-treated NETs, were cocultured with primary LECs for 24 h. **(A)** Schematic experimental design of NETs cocultured with LECs.** (B)** Illustration showing the isolation of polymorphonuclear leukocytes (PMNs) from mice by gradient centrifugation. **(C, D)** PMNs were identified by using Giemsa and IF staining. **(E)** SYTOX Orange staining showing nuclear membrane disruption and the subsequent release of DNA in PMA-treated PMNs. **(F)** LECs were identified by using microscopy and IF staining (VEGFR3). **(G)** The viability of LECs incubated with different concentrations of NETs for 24 h was determined by CCK-8 assays (n = 6, one-way ANOVA). **(H)** The viability of LECs incubated with NETs, PBS, or NETs+DNase I for 24 h was determined by CCK-8 assays (n = 6, one-way ANOVA). **(I)** The quantification of PI^+^ cells was performed using flow cytometry with Annexin V-FITC/PI staining. **(J)** The percentage of PI+ LECs in the different groups (n = 6, one-way ANOVA). **(K)** TUNEL staining was performed, and the number of TUNEL^+^ cells was determined. **(L)** The percentages of TUNEL^+^ LECs in the different groups (n = 6, one-way ANOVA). ns: P > 0.05, *P < 0.05, **p < 0.01. The data are the means ± SEMs.

Because NETs can induce EC damage 37, 52, we next examined whether NETs could damage LECs in vitro (Figure 8A). We used a coculture method to investigate the impact of NETs on LECs, and NETs were incubated with LECs. We obtained neutrophils from the peripheral blood of mice and verified them by Giemsa staining or Ly6g staining (Figure 8B-D). Then, the neutrophils were incubated with or without PMA. We found that PMA-stimulated neutrophils from mice exhibited NET formation (Figure 8E-right). VEGFR3 staining and imaging were used to identify LECs (Figure 8F). We confirmed that NETs exerted a dose-dependent cytotoxic effect on LECs. Our results revealed no significant difference in the viability of LECs treated with 500 ng/mL and 1000 ng/mL NETs. Therefore, for subsequent experiments, 1000 ng/mL NETs were used (Figure 8G). After 24 h of coculture, the viability of LECs was assessed using the CCK-8 assay. We observed a significant increase in cell viability in the DNase I-pretreated group compared to the untreated group (Figure 8H). Flow cytometry and propidium iodide (PI) staining revealed LEC impairment induced by NETs (Figure 8I), and pretreatment with DNase I could ameliorate this damage (Figure 8J). The same results were obtained by TUNEL staining (Figure 8K), and pretreatment with DNase I could reduce the number of TUNEL-positive cells (Figure 8L). Taken together, these findings suggest that NETs damage LECs in vitro, and pretreatment with DNase I could mitigate the detrimental effects of NETs on LECs.

### Quantitative transcriptome sequencing of LECs revealed leukocyte-mediated inflammatory RNA accumulation after coculture

**Figure 9:**
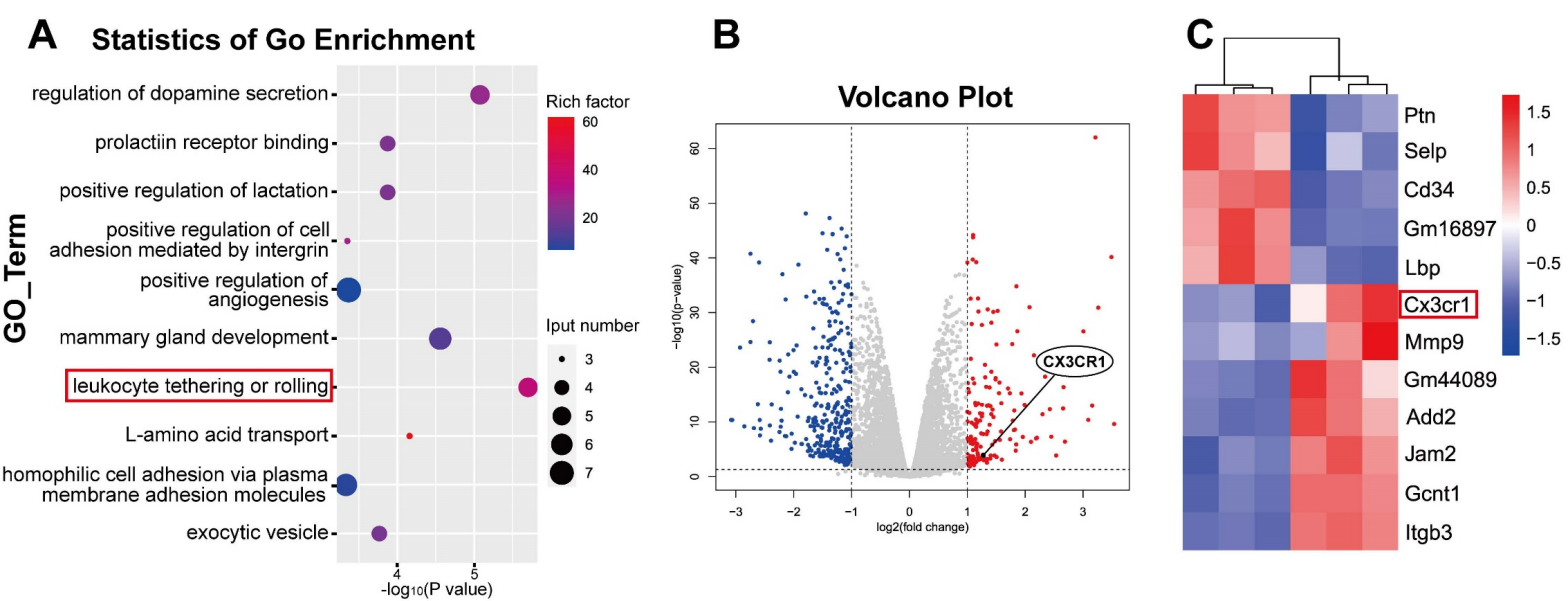
** Transcriptome sequencing analysis of injury pathways that were activated in LECs after being cocultured with NETs.** Following 24 h of coculture with 1000 ng/mL NETs, LECs were collected and subjected to RNA-Seq analysis according to the manufacturer's protocol.** (A)** GO enrichment of the transcriptome sequencing data showing that leukocyte tethering or rolling was upregulated in the LEC+NET group compared to the Control group.** (B)** A volcano plot showing the differentially expressed genes identified through transcriptome sequencing between the Control group and LEC+NET group. **(C)** Heatmap showing the top 12 genes related to leukocytes between the Control group and LEC+NET group.

Since the in vitro experiments demonstrated the direct LEC damage by NETs, our next objective was to elucidate the underlying molecular mechanisms by which NETs damage LECs. To investigate the mechanism of NET-induced LEC damage, we harvested LEC samples after 24 h of coculture with NETs. We subsequently performed bulk RNA sequencing of LECs and compared samples treated with NETs to untreated samples. Differential analysis of the entire gene set revealed a total of 480 genes that exhibited significant differential expression (logFC ≥ 1). We identified 153 upregulated genes and 327 downregulated genes in the NET-treated group compared to the untreated group (Figure 9B). Upregulated genes are represented in red, while downregulated genes are represented in blue. Furthermore, GO enrichment and pathway enrichment analyses were performed to explore the functional implications of these genes, and we observed pronounced activation of the leukocyte tethering or rolling pathway in LECs in response to NET treatment (Figure 9A). In-depth examination of the leukocyte tethering or rolling pathway highlighted the significant upregulation of Cx3cr1, Gcnt1, Add2, Jam2, and Gm44089. Considering that the injurious factor is NETs, we were particularly intrigued by leukocyte-associated genes among the differentially expressed genes. After screening for leukocyte-related differentially expressed genes, we observed that CX3CR1 ranked among the top 10 genes with the most significant alterations (Figure 9C). CX3CR1 is expressed in various tissues and plays a role in immune responses, inflammation, cell adhesion, and chemotaxis. Recent studies have indicated that CX3CR1 participates in the development and regulation of atherosclerosis 53. Given that Cx3cr1 is the primary receptor molecule that mediates leukocyte adhesion, we hypothesized that CX3CR1 was implicated in NET-mediated LEC damage. Subsequent experiments were performed to elucidate the underlying mechanisms of NET-induced LEC damage, with a specific focus on CX3CR1.

### Hydrocephalus, neurological impairment, and CSF drainage after IVH are ameliorated following CX3CR1 knockdown and in Cx3cr1^-/-^ mice

To investigate whether CX3CR1 is involved in NET-induced LEC damage in mice, we used AAV-mediated CX3CR1 knockdown via AAV-shRNA*cx3cr1* i.c.v. injection and established an IVH model 28 days after AAV i.c.v. injection.

**Figure 10:**
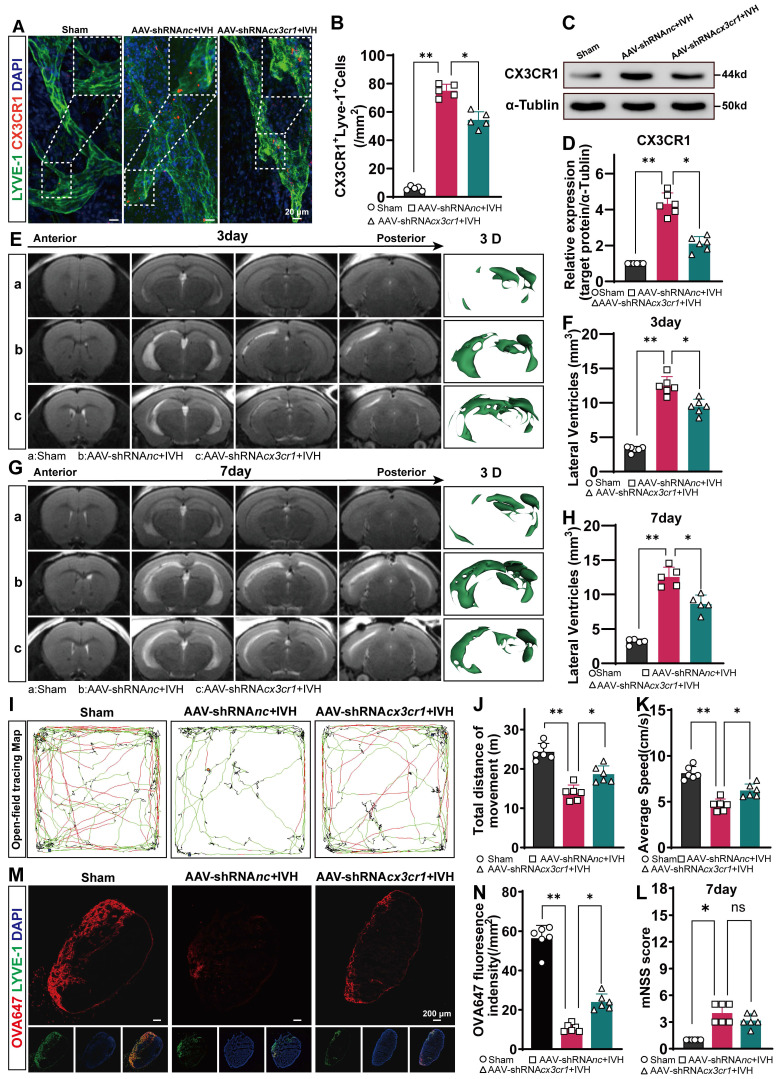
** Evaluation of hydrocephalus, neurological function and CSF drainage with or without CX3CR1 knockdown by AAV after IVH.** AAV-shRNA*cx3cr1* was engineered to knock down the expression of CX3CR1 in the mLVs of mice by i.c.v. injection. AAV-shRNA*nc* was injected as a control. Autologous blood IVH mice were generated 28 days after viral injection. **(A)** Representative photomicrographs of CX3CR1 and LYVE-1 staining in the TS of Sham, AAV-shRNA*nc+*IVH or AAV-shRNA*cx3cr1*+IHV groups 24 h after IVH. **(B)** Assessment of the number of CX3CR1^+^ LECs in the TS of the meninges (n = 5, one-way ANOVA).** (C)** Representative WB images of CX3CR1 in the meninges of mice in the Sham, AAV-shRNA*nc+*IVH and AAV-shRNA*cx3cr1*+IVH groups 24 h after IVH. ɑ-Tubulin was used as an internal reference protein. **(D)** Quantification of CX3CR1 expression (n = 6, one-way ANOVA). **(E, G)** T2-weighted images and three-dimensional reconstructions of the lateral ventricles were obtained three and seven days after IVH in the Sham group, AAV-shRNAnc+IVH group, and AAV-shRNAcx3cr1+IVH group and served as representative examples. **(F, H)** Quantification of the volume of lateral ventricles in each group (n = 6, 5, one-way ANOVA). **(I)** Representative open field movement trajectories of mice in the different groups were assessed 7 days after IVH. **(J)** The summary data show the distance traveled in the open field experiment (n = 6, one-way ANOVA).** (K)** Average speed of the different groups in the open field test (n = 6, one-way ANOVA). **(M)** Representative photomicrographs of LYVE-1-stained dCLNs in Sham, AAV-shRNA*nc*+IVH and AAV-shRNA*cx3cr1+*IVH mice were captured 2 h after i.c.m. injection of OVA647 (n = 6). **(N)** The mean fluorescence intensity of OVA647 in dCLNs was quantified (n = 6, one-way ANOVA). **(L)** mNSSs of the different groups (n = 6, non-parametric tests). ns: P > 0.05, *P < 0.05, **p < 0.01. The data are means ± SEMs.

**Figure 11:**
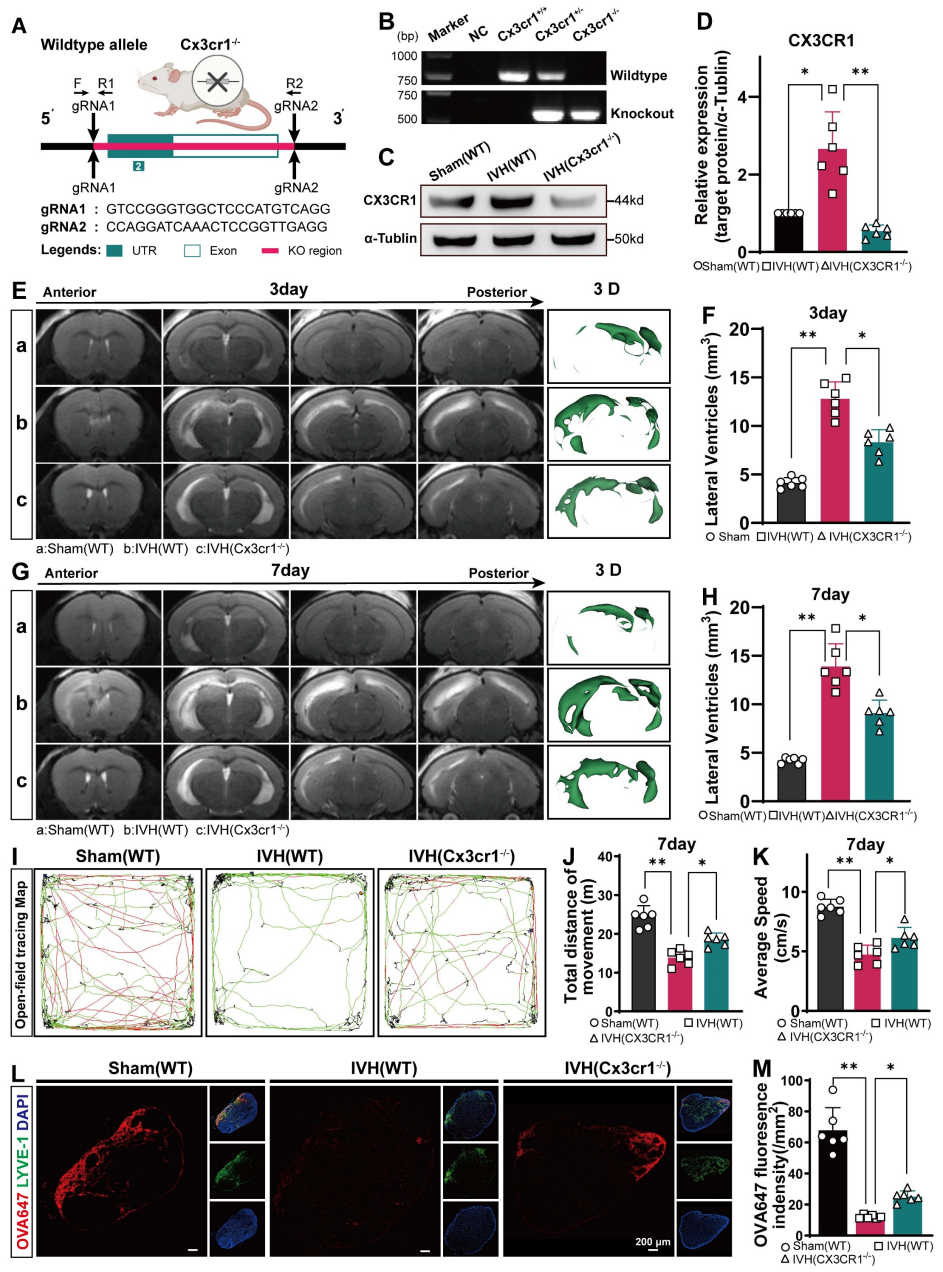
** Evaluation of hydrocephalus, neurological function and CSF drainage in Cx3cr1^-/-^ mice and control mice after IVH.** Cx3cr1^-/-^ mice were generated using CRISPR/Cas9. **(A)** Schematic diagram depicting the process used to generate Cx3cr1^-/-^ mice. **(B)** Mouse genotyping by PCR. F\R1 indicates for knockout sequence, and F\R2 indicates the wild-type sequence.** (C)** Representative WB images of CX3CR1 in the meninges of mice in the Sham, IVH (WT) and IVH (Cx3cr1^-/-^) groups 24 h after IVH. α-Tubulin was used as an internal reference protein. **(D)** Quantification of CX3CR1 expression (n = 6, one-way ANOVA). **(E, G)** Representative T2-weighted images and three-dimensional reconstructions of the lateral ventricles three and seven days after IVH in the Sham group, IVH (WT) group, and IVH (Cx3cr1^-/-^) group. **(F, H)** Quantification of the volume of lateral ventricles in each group (n = 6, one-way ANOVA). **(I)** Representative movement trajectories of mice in the different groups in the open field test 7 days after IVH. **(J)** The summary data show the distance traveled in the open field experiment (n = 6, one-way ANOVA).** (K)** Average speed of the different groups in the open field test (n = 6, one-way ANOVA). **(L)** Representative photomicrographs of LYVE-1-stained dCLNs in Sham, IVH (WT) and IVH (Cx3cr1^-/-^) group mice were captured 2 h after i.c.m. injection of OVA647. **(M)** The mean fluorescence intensity of OVA647 in dCLNs was quantified (n = 6, one-way ANOVA). *P < 0.05, **p < 0.01. The data are means ± SEMs.

We examined the changes in mLVs by immunostaining with antibodies against LYVE-1 and CX3CR1 to determine the number of CX3CR1^+^ in LYVE-1 cells in the meningeal lymphatic system. Our findings revealed that CX3CR1 was present within LYVE-1**-**positive mLVs (Figure 10A). The number of CX3CR1^+^ cells was reduced in the AAV-shRNA*cx3cr1+*IVH group compared to the AAV-shRNA*nc+*IVH group (Figure 10B). The Western blot results showed that the expression of CX3CR1 was altered in the meninges in the AAV-shRNA*cx3cr1+*IVH group compared with the AAV-shRNA*nc+*IVH group (Figure 10C-D). These results indicate successful knockdown of CX3CR1 mediated by AAV in the mLVs of mice. Moreover, we employed CRISPR/Cas9 technology to generate CX3CR1^-/-^ mice and conducted further validation experiments (Figure 11A). Deletion of the mouse CX3CR1 gene was subsequently confirmed through PCR analysis (Figure 11B). The Western blot results demonstrated a significant reduction in the expression of CX3CR1 in the meninges of the IVH (Cx3cr1^-/-^) group compared to that of the IVH (WT) group and Sham (WT) group (Figure 11C-D). These results indicate successful knockout of CX3CR1 in Cx3cr1^-/-^ mice.

Next, we examined whether hydrocephalus after IVH could be alleviated by knockdown of CX3CR1 and in Cx3cr1^-/-^ mice. We evaluated the degree of hydrocephalus in the Sham, AAV-shRNA*nc+*IVH, and AAV-shRNA*cx3cr1+*IVH groups by assessing lateral ventricular volumes according to T2-weighted imaging and 3D reconstruction images 3 and 7 days after IVH. We found that the AAV-shRNA*nc+*IVH group had more lateral ventricular dilation than the AAV-shRNA*cx3cr1+*IVH group (Figure 10 E, G). Both groups exhibited severe hydrocephalus 3 days after hemorrhage, which persisted until 7 days (Figure 10F, H). In comparison to the AAV-shRNA*cx3cr1+*IVH group, the AAV-shRNA*nc+*IVH group exhibited a significant increase in lateral ventricular volume, suggesting that prior knockdown of CX3CR1 could improve hydrocephalus after IVH. In the experimental with Cx3cr1^-/-^ mice, we obtained similar results, demonstrating that the degree of lateral ventricular dilation was reduced in the IVH (Cx3cr1^-/-^) group compared to the IVH (WT) group (Figure 11 E-H). These results suggest that hydrocephalus following IVH can be ameliorated by CX3CR1 knockdown and in Cx3cr1^-/-^ mice.

We examined whether motor function after IVH could be improved by knockdown of CX3CR1 prior to IVH and in Cx3cr1^-/-^ mice. Motor function was assessed using the open field test on day 7 after IVH (Figure 10I). Compared with the Sham group, the AAV-shRNA*nc*+IVH group exhibited markedly impaired motor function, including total movement and average speed, on day 7 after IVH (Figure 10J-K). Interestingly, prior knockdown of CX3CR1 in mice promoted locomotion recovery, whereas mice without CX3CR1 knockdown exhibited no changes in functional recovery (Figure 10J, K). In comparison to the AAV-shRNA*nc+*IVH group, the AAV-shRNA*cx3cr1*+IVH group exhibited superior locomotion recovery, suggesting that prior knockdown of CX3CR1 could improve motor function after IVH. Neurological deficits were evaluated using the mNSS scale on day 7 post-IVH. Overall, neurological deficits were not ameliorated in the AAV-shRNA*cx3cr1*+IVH group compared with the AAV-shRNA*nc*+IVH group (Figure 10L). In the experiment with Cx3cr1^-/-^ mice, we found that Cx3cr1^-/-^ improved motor function after IVH (Figure 11I-K). These results suggest that motor function following IVH is enhanced by CX3CR1 knockdown and in Cx3cr1^-/-^ mice. Then, we examined whether could mLV drainage after IVH could be improved by prior knockdown of CX3CR1 and in Cx3cr1^-/-^ mice. OVA647 was injected i.c.m. into mice with or without CX3CR1 knockdown, and the presence of OVA647 in draining dCLNs was examined for 2 h (Figure 10M). Our results showed that prior knockdown of CX3CR1 promoted OVA647 drainage in dCLNs after IVH. Conversely, IVH mice without CX3CR1 knockdown showed no effects on the drainage of OVA647 (Figure 10N). In the experiment with Cx3cr1^-/-^ mice, we also found that the Cx3cr1^-/-^ promoted OVA647 drainage after IVH (Figure 11 L-M). These results suggest that the drainage of mLVs after IVH can be improved by prior knockdown of CX3CR1 and in Cx3cr1^-/-^ mice.

### LEC damage is alleviated after CX3CR1 knockdown and in Cx3cr1^-/-^ mice

**Figure 12:**
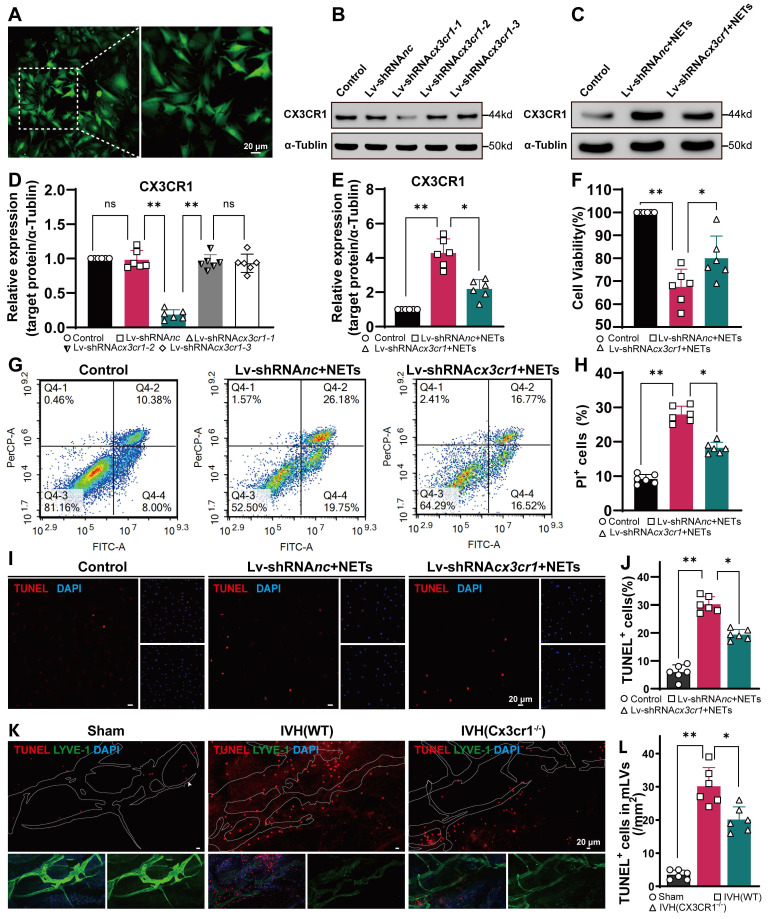
** Evaluating the role of CX3CR1 in NET-induced LEC damage in vitro and in vivo.** Primary LECs were transfected with a lentivirus (Lv-shRNA*cx3cr1,* Lv-shRNA*nc*) to knockdown CX3CR1. Following successful transfection, subsequent experiments were conducted on LECs. After LECs and NETs were cultured for 24 h, LECs were collected and analyzed. **(A)** Representative images showing successful LV transfection in LECs.** (B)** Three target sequences were designed to knockdown CX3CR1, and the specificity and efficacy of LV-mediated knockdown of CX3CR1 were confirmed by WB analysis. α-Tubulin was used as an internal reference protein. **(C)** Representative WB images of CX3CR1 in LECs in the Control, (LECs+Lv-shRNA*nc*)+NET and (LECs+Lv-shRNA*cx3cr1*)+NET groups 24 h after coculture.** (D, E)** Quantification of CX3CR1 expression (n = 6, one-way ANOVA). **(F)** The viability of LECs in the different groups after coculture with NETs or PBS for 24 was determined by CCK-8 assays (n = 6, one-way ANOVA).** (G)** Annexin V-FITC/PI staining and flow cytometry were used to determine the percentage of PI^+^ cells. **(H)** Quantification of the percentage of PI^+^ LECs in the different groups (n = 6, one-way ANOVA). **(I)** TUNEL^+^ cells were measured by TUNEL staining. **(J)** Quantification of the percentage of TUNEL^+^ LECs in the different groups (n = 6, one-way ANOVA). **(K)** Representative images showing TUNEL and LYVE-1 staining in the TS of Sham, IVH (WT), or IVH (Cx3cr1^-/-^) group mice at 24 h after IVH. **(L)** The number of TUNEL-positive LECs surrounding the TS in the meninges of mice (n = 6, one-way ANOVA). ns: P > 0.05, *P < 0.05, **p < 0.01. The data are means ± SEMs.

To investigate whether CX3CR1 is critically involved in NET-induced LEC damage in vitro, we performed Lv-mediated CX3CR1 knockdown in LECs via Lv-shRNA*cx3cr1* transduction. Subsequently, the LECs with or without CX3CR1-knockdown were cocultured with NETs. The results revealed successful lentiviral transduction of the cells (Figure 12A).

To ensure optimal knockdown of CX3CR1, we designed and constructed three lentiviral sequences. WB analysis revealed that Lv-shRNA*cx3cr1* exhibited the most efficient knockdown effect (Figure 12B, D). Then, we measured the expression levels of CX3CR1 by WB analysis in each group with or without NETs. CX3CR1 expression was lower in LECs with prior knockdown of CX3CR1 after 24 h of coculture with NETs than in the Control group without knockdown (Figure 12C, E). After 24 h of coculture, the viability of LECs was assessed using the CCK-8 assay. We observed a significant increase in cell viability in the CX3CR1 knockdown group compared to the group without knockdown (Figure 12F). Flow cytometry and propidium iodide (PI) staining revealed LEC impairment induced by NETs in each group (Figure 12G), and CX3CR1 knockdown could ameliorate the damage (Figure 12H). The same results were obtained by TUNEL staining (Figure 12I), and prior knockdown of CX3CR1 could reduce the number of TUNEL-positive cells (Figure 12J). Taken together, these findings suggest that NETs damage LECs in vitro, and prior knockdown of CX3CR1 can mitigate the detrimental effects of NETs on LECs.

To explore the critical role of CX3CR1 in NET-induced LEC damage in vivo, we assessed the extent of LEC damage in Cx3cr1^-/-^ mice following IVH. TUNEL staining revealed that the IVH (Cx3cr1^-/-^) group exhibited a reduced number of TUNEL-positive cells compared to the IVH (WT) group (Figure 12K, L). Taken together, these findings suggest that NET-induced LEC damage could be mitigated by CX3CR1 knockdown and in Cx3cr1^-/-^ mice.

## Discussion

CSF blockages are significant neurological complications following ICH/IVH, and they can directly contribute to the development of brain injury and hydrocephalus 7, 54. However, the precise mechanism underlying these blockades remains incompletely understood 55. Early perspectives in the field are that the choroid plexus is responsible for the production of the majority of CSF, which then circulates through the ventricles, cisterns, and subarachnoid space before being absorbed into the bloodstream by the arachnoid villi 20. Recently, the presence and role of the meningeal lymphatic system in the brain have been proposed to facilitate the transport of CSF components, immune cells, and metabolic byproducts from the CNS to CLNs under diverse pathological conditions, such as Alzheimer's disease, multiple sclerosis, and stroke 21-23, 25. Here, we provided evidence that mLVs are involved in the pathogenesis of hydrocephalus and brain injury following IVH. NET degradation can improve hydrocephalus and neurologic dysfunction after IVH. Additionally, our results suggested that NET degradation improved the ability of mLVs to drain biological molecules after IVH. Moreover, NET degradation could ameliorate mLV damage. Taken together, our study revealed the involvement of the meningeal lymphatic system in the development of hydrocephalus and subsequent IVH. Furthermore, we demonstrated that NETs could damage the meningeal lymphatic system after IVH. Importantly, our findings suggest that inhibiting NETs could protect against hydrocephalus and neurological damage in the context of IVH.

NETs were initially described in 2004. Previous studies have demonstrated the occurrence of NET formation in arterial thrombi following acute cerebral ischemia. In our previous study, significant neutrophil infiltration and NET formation were observed following IVH 38. Subsequently, in our study on SAH, we observed the colocalization of NETs with fibrinogen-positive microthrombi 29. Here, we have provided evidence of the presence of NETs in the meningeal lymphatic system following IVH. TEM revealed discontinuous junctions that confirmed the permeability of dural blood and lymphatic vessels for fluid and cell exchange. Additionally, CFSE-labeled neutrophils with complete cell morphology were observed within LYVE-1^+^ mLVs and dCLNs. Recent studies have shown that NETs induce fibrin deposition in the lungs and local lymph nodes of patients infected with COVID-19 30. Lymphatic thrombosis has also been reported in various diseases, such as cancer, infections, amyloidosis, and lymph node dissection 31. Importantly, the deposition of RBCs, leukocytes, and fibrin within mLVs was discovered by SEM following IVH. Our findings further revealed that NET degradation ameliorated hydrocephalus and improved neurological deficits during this phase. Taken together, these findings suggest that NETs may induce fibrin deposition, leading to lymphatic thrombosis and subsequent dysfunction of mLVs, ultimately resulting in hydrocephalus.

Recent studies have indicated that NETs can directly or indirectly cause EC damage, and their involvement has been reported in diabetic kidney disease and systemic lupus erythematosus (SLE) 37, 52. We provided evidence that NETs damage LECs in vitro, and pretreatment with DNase I mitigated the detrimental effects of NETs on LECs in vitro. Moreover, the WB results demonstrated increased expression of lymphangiogenesis markers such as FOXC2, VEGFC, PROX1, VE-cadherin and LYVE-1 in the meninges of the DNase I-treated group compared with the IVH group at 24 h. We found a significant reduction in TUNEL-positive cells in mLVs in the DNase I-treated group compared to the IVH group. Taken together, these data suggest that the DNase I treatment can improve meningeal lymphatic endothelial activity by improving the morphology, production, and damage to meningeal lymphatic endotheliocytes after IVH, suggesting that DNase I treatment may promote the recovery of injured LECs following IVH.

CX3CR1 is expressed mainly in certain leukocyte populations and is typically low in healthy blood vessels. However, CX3CR1 expression is significantly increased in pathological conditions such as atherosclerotic plaques 56. RT‒PCR and WB analysis demonstrated the presence of CX3CR1 mRNA and protein in human umbilical vein and coronary artery ECs, and increased expression of CX3CR1 in ECs enhanced the adhesion of human neutrophils to ECs 57. Recent studies have shown that chronic hypoxia in wounds leads to increased expression of CX3CR1 in ECs, resulting in impaired angiogenesis. Targeting CX3CR1 can improve damage in chronic wounds 58. Transcriptome sequencing and analysis of differential gene expression showed significant activation of the leukocyte adhesion and rolling pathway following the coculture of LECs with NETs. Further investigation revealed that CX3CR1 may play a crucial role in this pathway. Immunohistochemical staining and WB analysis demonstrated increased expression of CX3CR1 in mLVs after IVH. We designed AAVs and lentiviruses to knock down CX3CR1 and produced Cx3cr1^-/-^ mice via genetic engineering. T2-weighted imaging suggested that hydrocephalus after IVH was ameliorated by CX3CR1 knockdown and in Cx3cr1^-/-^ mice. Behavioral analyses indicated that motor function after IVH was improved by CX3CR1 knockdown and in Cx3cr1^-/-^ mice. Injection of OVA647 into the cisterna magna suggested that the drainage of mLVs after IVH was enhanced by prior knockdown of CX3CR1 and in Cx3cr1^-/-^ mice. Our findings suggested that in vitro, NETs damage LECs, and prior knockdown of CX3CR1 could mitigate the detrimental effects of NETs on LECs. In vivo experiments revealed similar results, demonstrating that NET-mediated LEC injury was alleviated in Cx3cr1^-/-^ mice. In summary, these findings collectively suggest that following IVH, NETs upregulate CX3CR1 expression on LECs, facilitating the recruitment and adhesion of leukocytes. The recruitment of leukocytes exacerbates local inflammatory responses and promotes further formation of NETs. The increased presence of NETs in mLVs can directly damage LECs and induce fibrin deposition, leading to lymphatic thrombosis and subsequent disruption of meningeal lymphatic system function.

This study has limitations. One of the primary limitations is that our hypothesis posits that NETs induce the upregulation of CX3CR1, leading to neutrophil accumulation and increased NET formation, thereby exacerbating damage. However, the point at which this feedback loop reaches equilibrium remains unclear. We hypothesize that equilibrium is achieved when mLVs are completely obstructed, preventing further neutrophil accumulation. This hypothesis, however, requires further experimental validation. In addition, in this study, we observed that NETs induced LEC damage via CX3CR1, but the specific role and mechanism of CX3CR1 in NET-induced LEC damage were not fully elucidated. Considering that NETs are complex structures, we hypothesize that CX3CR1 plays a pivotal role in the cascade of events leading to NET-induced LEC damage. Recent studies of diabetic kidney disease suggested that NETs may induce glomerular EC (GEC) pyroptosis through the surface charge of the cell membrane 37. Similarly, studies of SLE showed that small RNAs externalized in NETs could induce EC damage 52. Future studies investigating molecular mechanisms via biochemical and genetic strategies will further our understanding of the genes and signaling pathways that are essential for NET-induced LEC damage by CX3CR1. Moreover, the IVH model we employed was induced by direct injection of autologous blood into the ventricles but did not simulate blood entry into the ventricles after ICH; furthermore, we did not consider the source of the bleeding (e.g., the basal ganglia, frontal lobe, hippocampus, cortex, etc.) 59. It is possible that failing to control for these differences in our model resulted in the lack of difference in mNSSs between the vehicle and treatment groups, despite the widespread application of the mNSS scale for neurological functional assessment after ICH. Finally, we used knockdown or knockout approaches but did not use more advanced methods, such as conditional knockout in mice. In future studies, we plan to use conditional knockout mice to further validate our findings.

## Conclusion

The meningeal lymphatic system participates in the formation of secondary hydrocephalus and brain injury after IVH. Following IVH, there was significant accumulation of neutrophils, NETs, and fibrin in the meningeal lymphatic system. This resulted in acute injury in LECs and the occurrence of lymphatic thrombosis. CX3CR1 is a key molecule in NET-induced endothelial damage and lymphatic thrombosis, leading to the dysfunction of mLVs and exacerbating hydrocephalus and brain injury. NETs may be a critical target for addressing the obstruction of meningeal lymphatic drainage after IVH.

## Supplementary Material

Supplementary figures and tables.

## Figures and Tables

**Figure 13:**
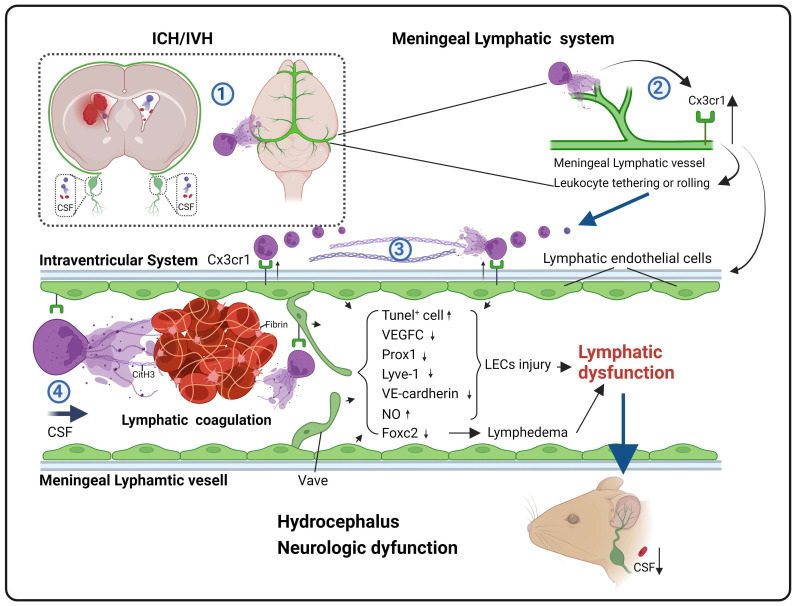
** A schematic diagram illustrating the underlying mechanism.** After IVH, NETs were induced in the hematoma, ventricular system and mLVs. Next, NETs upregulated CX3CR1 expression in mLVs, which recruited more neutrophils to form NETs and led to lymphatic thrombosis and LEC damage.

## Abbreviations

ICH: intracerebral hemorrhage
IVH: intraventricular hemorrhage
NETs: neutrophil extracellular traps
CSF: cerebrospinal fluid
CLNs: cervical lymph nodes
mLVs: meningeal lymphatic vessels
RBCs: red blood cells
dCLNs: deep cervical lymph nodes
sCLNs: superficial cervical lymph nodes
LEC: lymphatic endothelial cell
MRI: magnetic resonance imaging
SEM: scanning electron microscopy
TEM: transmission electron microscopy
CFSE: carboxyfluorescein diacetate succinimidyl ester
COS: confluence of sinus
SSS: superior sagittal sinus
CitH3: citrullinated histone H3
NO: nitric oxide
OVA647: fluorescence-labeled ovalbumin conjugated to Alexa Fluor 647
PMA: phorbol 12-myristate 13-acetate
EVD: extraventricular drain
mNSS: modified Neurological Severity Score
