# Supplementary material for: Neutrophil extracellular trap-mediated impairment of meningeal lymphatic drainage exacerbates secondary hydrocephalus after intraventricular hemorrhage
Source: Theranostics. 2024 Feb 24;14(5):1909–38. doi: 10.7150/thno.91653 (PMC10945341; doi:10.7150/thno.91653)
Supplement: Supplementary file 1 — Supplementary figures and tables. [file thnov14p1909s1.pdf]

SUPPLEMENTAL MATERIALS

Tables S1. Primer sequences and conditions for RT-qPCR.

Target	Product length (bp)	Annealing temperature (℃)	Primer (5'-3')	Genbank accession no.
VEGFC-F	147	60	CTGCTGCACATTATAACACAGAGA	22341
VEGFC-R			ACGGACACACATGGAGGTTTAAA	
FOXC2-F	300	60	ACTCTTACGACTGCACCAAATACT	14234
FOXC2-R			TCAAACTGAGCTGCGGATAAGT	
GAPDH-F	150	60	ATGGGAAGCTGGTCATCAAC	14433
GAPDH-R			TGCTGTAGCCAAATTCGTTG	

Table S2. Demographics and baseline data of Control and ICH/IVH

	Control(n=9)	ICH/IVH(n=9)	Fisher/t	*P*
Sex male, n (%)	5（55.5）	4 (44.5)	-	0.999
Age ($\overline{X}$±S, years)	52.6±9.6	67±15	t=2.43	0.027
Education			-	0.999
Postgraduate, n (%)	1（11.1）	2（22.2）		
Undergraduate, n (%)	2（22.2）	1（11.1）		
Secondary school, n (%)	4（44.4）	3（33.3）		
Primary school, n (%)	2（22.2）	3（33.3）		
Psychological consultation, n (%)	0（0）	0（0）	-	—
Antidepressant, n (%)	0（0）	0（0）	-	0.999
Parkinson's disease, n (%)	0（0）	0（0）	-	0.999
Huntington, n (%)	0（0）	0（0）	-	0.999
Alzheimer's disease, n (%)	0（0）	0（0）	-	0.999
Hypertension, n (%)	1（11.1）	8（88.8）	-	＜0.001
Hyperlipidemia, n (%)	0（0）	1（11.1）	-	0.999
Diabetes, n (%)	0（0）	0（0）	-	0.999
Warfarin treatment, n (%)	0（0）	1（11.1）	-	0.999
History of stroke, n (%)	0（0）	0（0）	-	0.999
Smoking, n (%)	2（22.2）	4（44.4）	-	0.620
Alcohol abuse, n (%)	1（11.1）	3（33.3）	-	0.576

Note:- stands for fisher exact probaliity method, t stands for Student's t test. ICH: intracerebral hemorrhage. IVH：intraventricular hemorrhage.

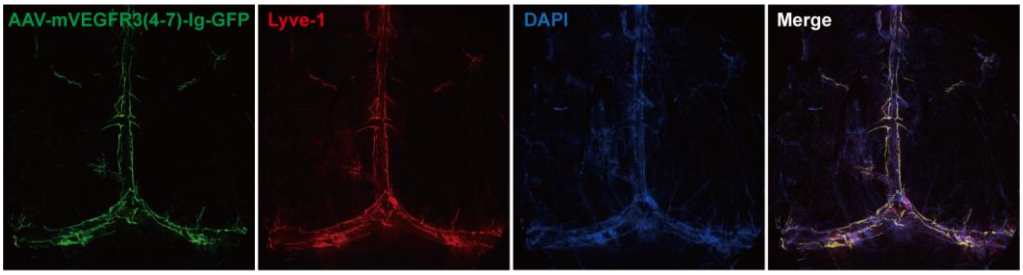

Figure S1. Representative photomicrographs of LYVE1-stained mLVs were obtained four weeks later, following the i.c.v. injection of the specified AAVs into six-week-old mice.

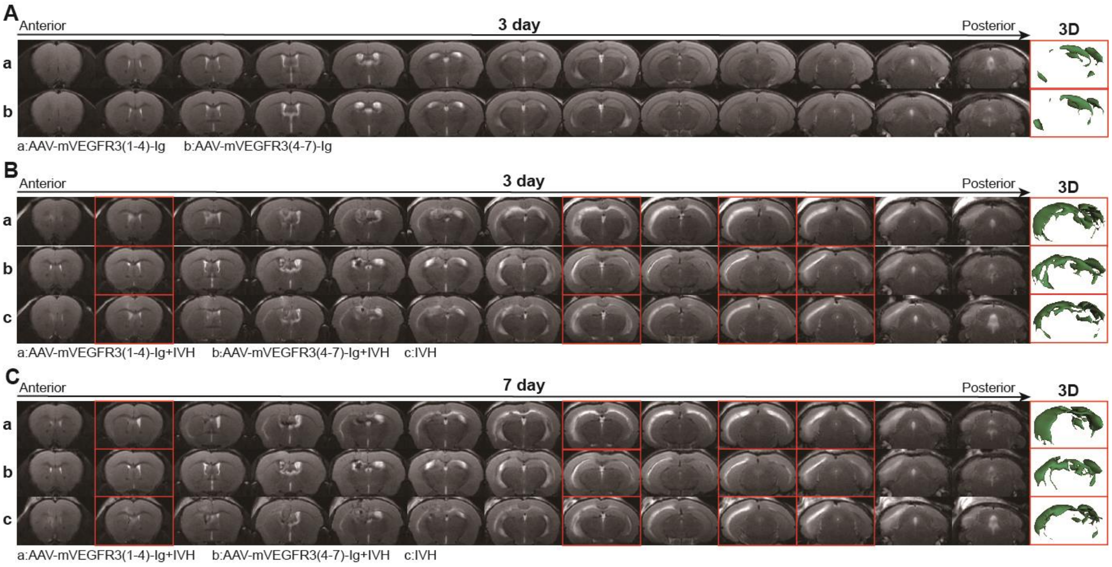

Figure S2. (A) All T2-weighted MRI images and 3D reconstructions of the lateral ventricles were acquired on 4 weeks after AAV-mVEGFR3(1-4)-Ig and AAV-mVEGFR3(4-7)-Ig injection. **(B,C)** Representative all T2-weighted images along with 3D reconstructions of lateral ventricles, captured on days 3 and 7 following IVH, from the AAV-mVEGFR3-Ig(1-4)+IVH group, AAV-mVEGFR3(4-7)-Ig+IVH group, and IVH group.

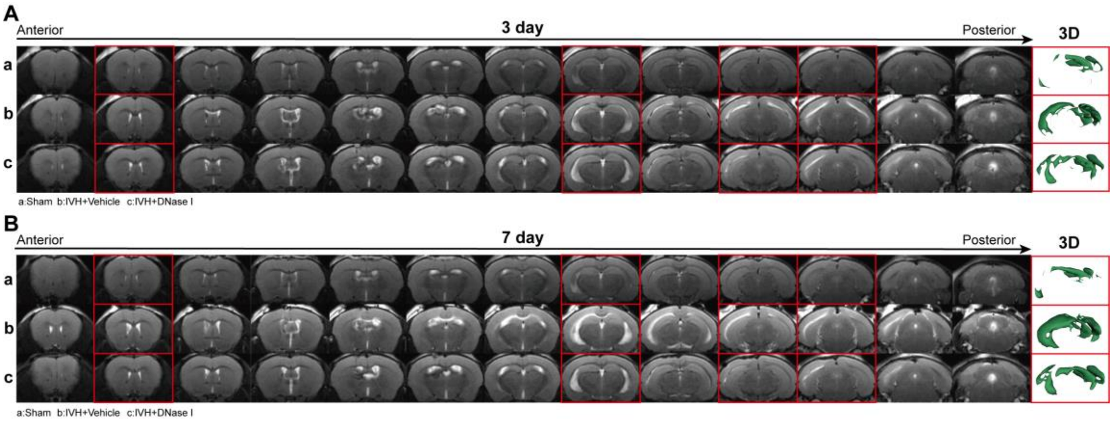

Figure S3. (A) All T2-weighted MRI images and 3D reconstructions of the lateral ventricles were acquired on three days for the Sham, IVH, and IVH+DNase I groups on days 3. **(B)** All T2-weighted MRI images and three-dimensional reconstructions of the lateral ventricles were acquired on seven days and for the Sham, IVH, and IVH+DNase I groups on days 7 after IVH.

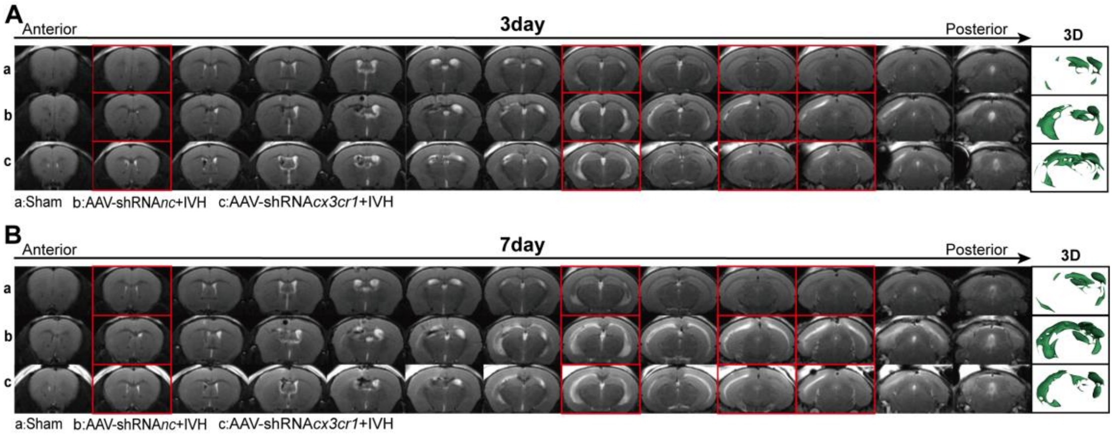

Figure S4. (A) All T2-weighted MRI images and 3D reconstructions of the lateral ventricles were acquired on three days for the Sham, AAV-shRNA*nc*+IVH, and AAV-shRNA*cx3cr1*+IVH groups on days 3 after IVH. **(B)** All T2-weighted MRI images and three-dimensional reconstructions of the lateral ventricles were acquired on seven days for the Sham, AAV-shRNA*nc*+IVH, and AAV-shRNA*cx3cr1*+IVH group on days 7 after IVH.

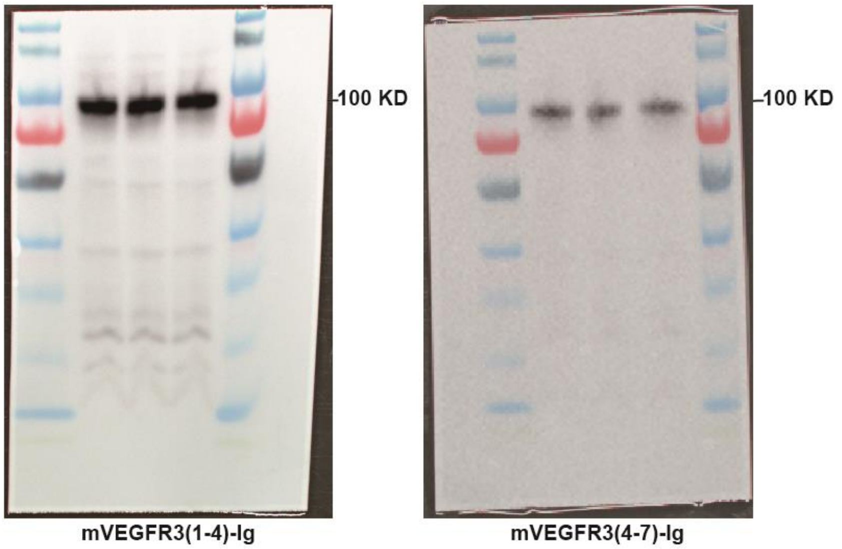

Figure S5. Western blot analysis revealing mVEGFR3-Ig protein in the serum one week following an i.p. AAV injection is shown.

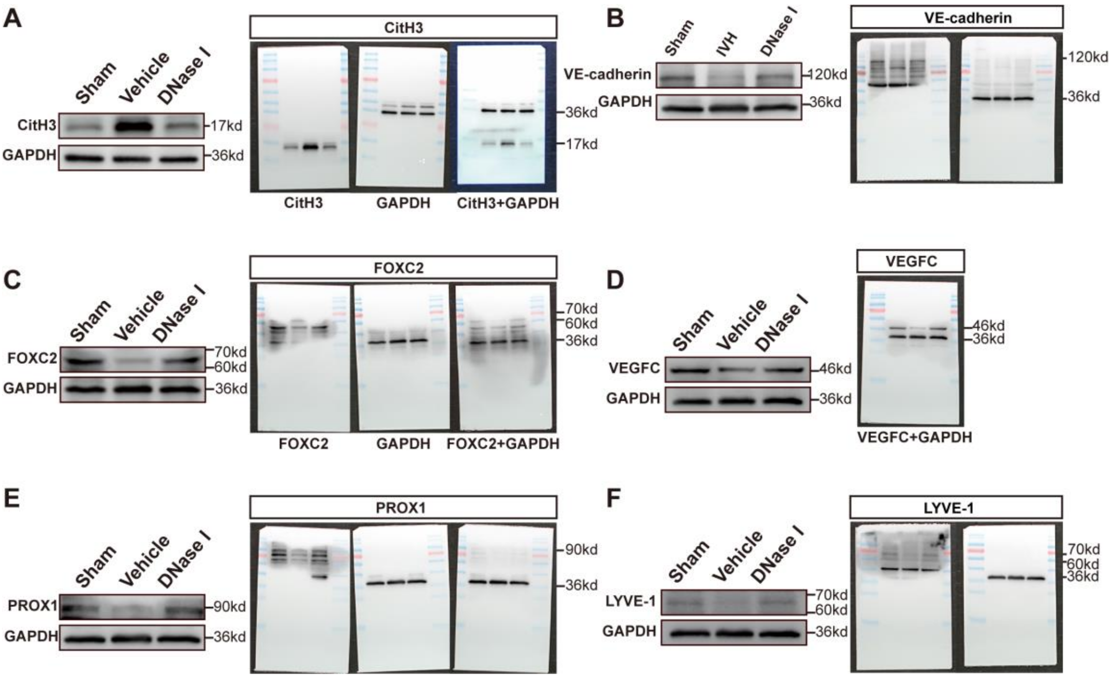

Figure S6. (A) A Western blot analysis revealing CitH3 protein in meninges at 24 h after IVH. GAPDH was used as an internal reference protein. **(B)** A Western blot analysis revealing VE-cadherin protein in meninges at 24 h after IVH. **(C)** Western blot analysis revealing FOXC2 protein in meninges at 24 h after IVH. **(D)** Western blot analysis revealing VEGFC protein in

meninges at 24 h after IVH. **(E)** Western blot analysis revealing PROX1 protein in meninges at 24 h after IVH. **(F)** Western blot analysis revealing LYVE-1 protein in meninges at 24 h after IVH.

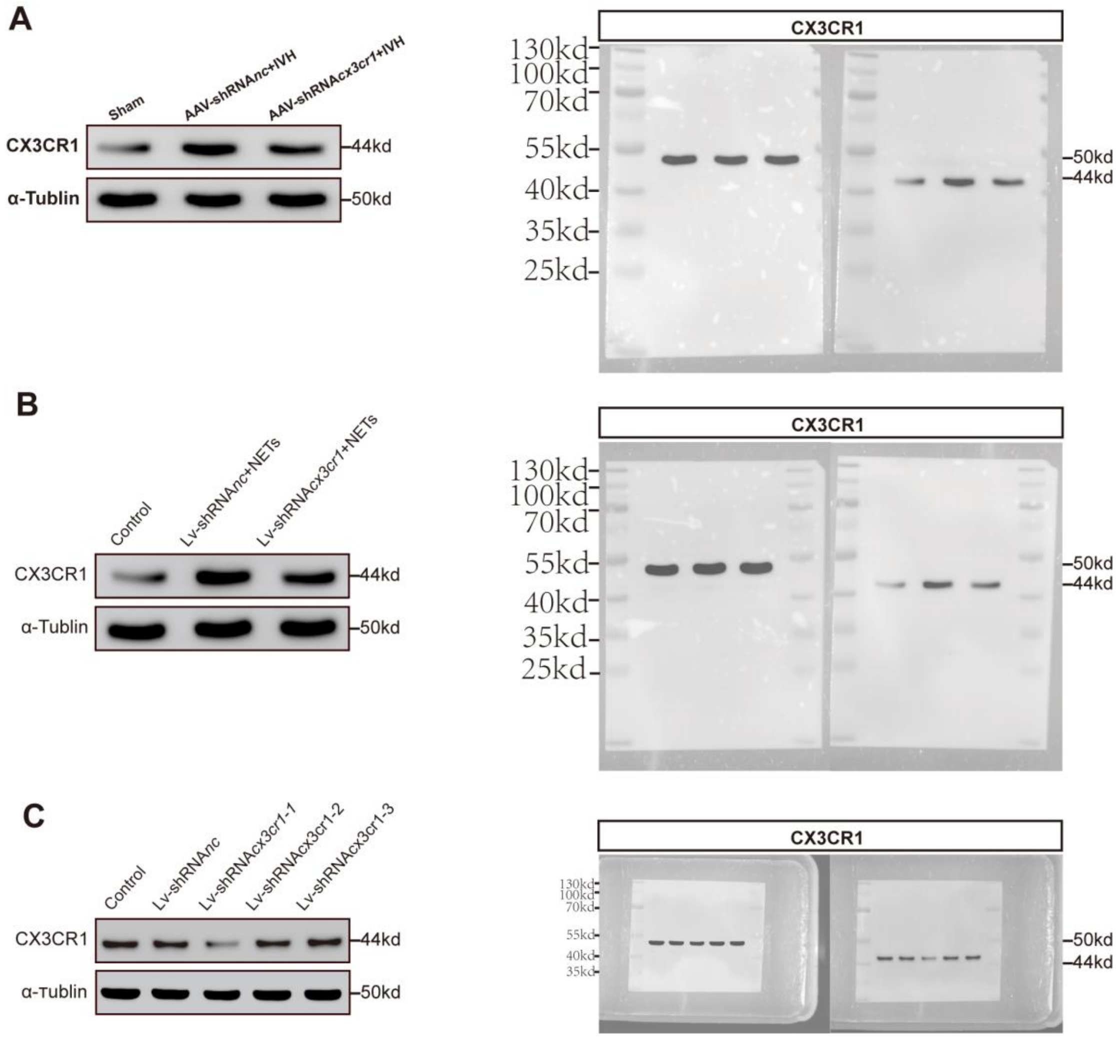

Figure S7. (A) Representative western blot images of CX3CR1 in the meninges of mice in the sham, AAV-shRNA*nc*+IVH and AAV-shRNA*cx3cr1*+IVH groups at 24 h after IVH. α-Tublin was used as an internal reference protein. **(B)** Representative WB images of CX3CR1 in LECs in the Control, (LECs+Lv-shRNA*nc*)+NET and (LECs+Lv-shRNA*cx3cr1*)+NET groups 24 h after coculture. **(C)** Three target sequences were designed to knockdown CX3CR1, and the specificity and efficacy of LV-mediated knockdown of CX3CR1 were confirmed by WB analysis. α-Tubulin was used as an internal reference protein.

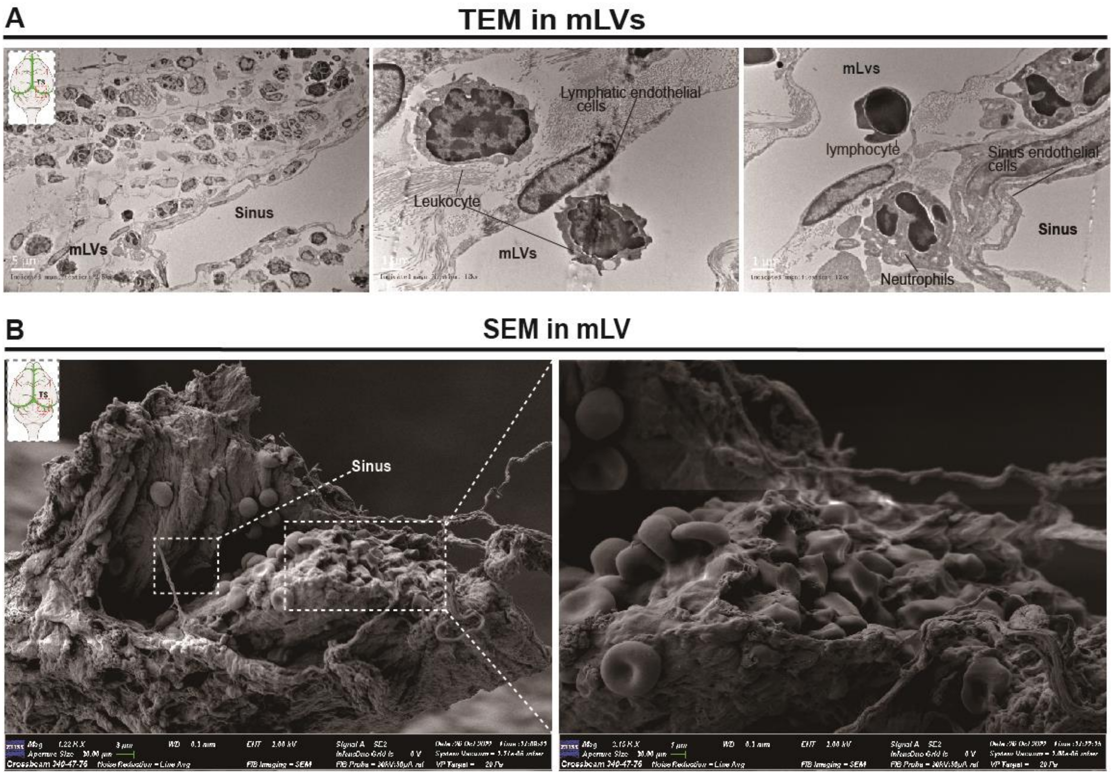

Figure S8. (A) Transmission electron microscopy (TEM) revealing the ultrastructural composition of the TS, including sinus endothelial cells, lymphatic endothelial cells, lymphocytes, neutrophils, and leukocyte interactions after 24 h of IVH. **(B)** Scanning electron microscopy (SEM) revealing the ultrastructural composition of the TS, including the sinus, mLVs, and entrainment of erythrocytes, polymorphonuclear neutrophils, and fibrin within the mLVs following post-IVH drainage.

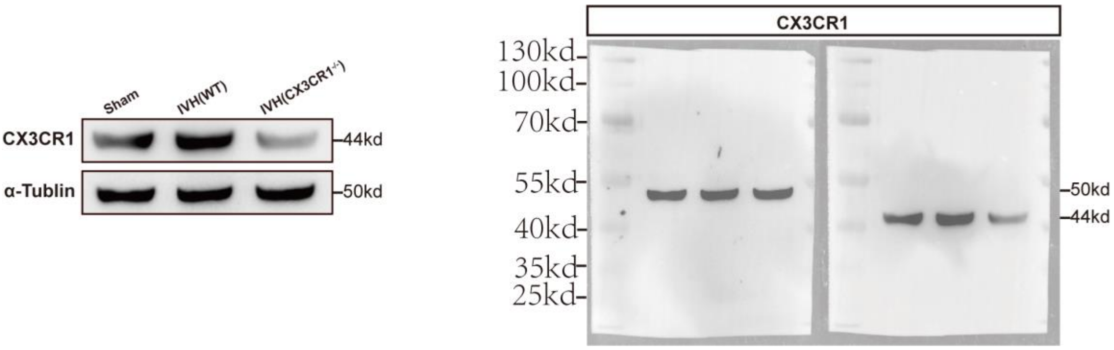

Figure S9. Western blot analysis revealing CX3CR1 protein in the meninges of mice in the Sham,

IVH(WT) and IVH($CX3CR1^{-/-}$) groups at 24 h after IVH.

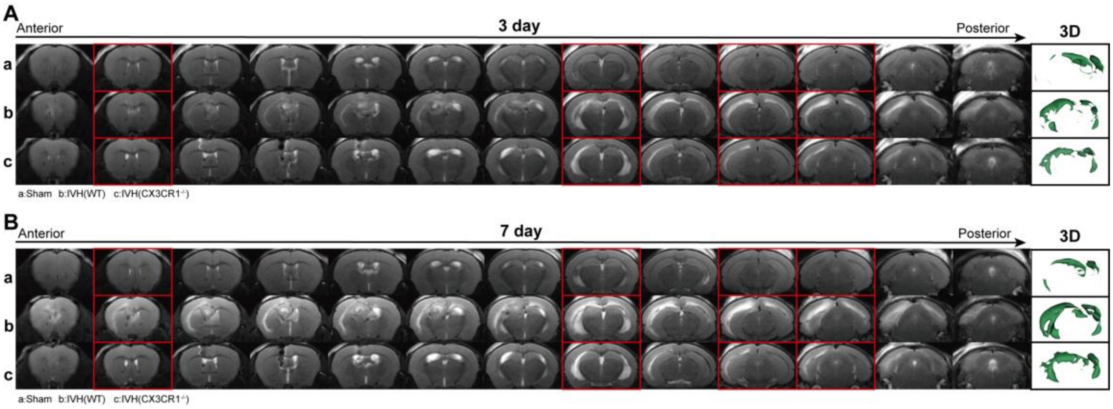

Figure S10. (A) All T2-weighted MRI images and 3D reconstructions of the lateral ventricles were acquired on three days for the Sham, IVH(WT), and IVH($CX3CR1^{-/-}$) groups. **(B)** All T2-weighted MRI images and three-dimensional reconstructions of the lateral ventricles were acquired on seven days for the Sham, IVH(WT), and IVH($CX3CR1^{-/-}$) groups.

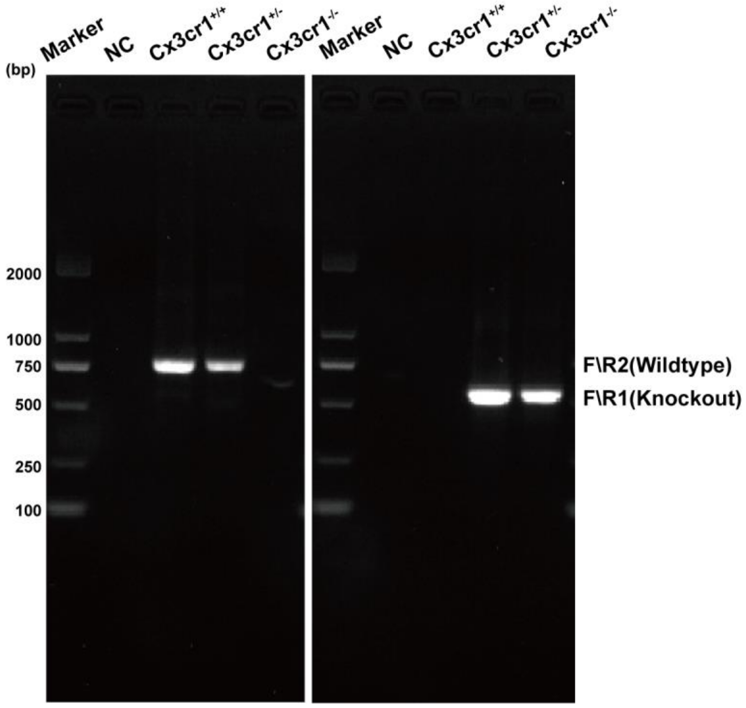

Figure S11. The PCR identification of mouse genotypes. F\R1 is used for knockout sequence and F\R2 for wildtype sequence.

Supplemental Methods

Experiments design and grouping:

The detail of the animals used was shown as follow: Total WT mice: 351 (0.8% mortality); Total Cx3cr1$^{-/-}$ mice: 18 (0 mortality).

Experiment 1: Western blots (WB), Immunofluorescence staining (IF), Hydrocephalus and RNA sequencing (RNA-seq).

Tests	AAV-mVEGFR3-Ig(1-4)(WT)	AAV-mVEGFR3-Ig(4-7)(WT)	AAV-mVEGFR3-Ig(1-4)+IVH(WT)	AAV-mVEGFR3(4-7)+IVH(WT)	IVH(WT)
WB	3	3	0	0	0
RNA-seq	0	0	3	3	0
IF	6	6	0	0	0
MRI	6	6	6	6	6

(Notes: Number of mice; Total number: 42.)

Experiment 2: Immunofluorescence staining (IF), scanning electron microscopy (SEM) and transmission electron microscopy (TEM).

Tests	IVH(WT)
IF	12
SEM	3
TEM	3

(Notes: Number of mice; Total number: 18)

Experiment 3: Immunofluorescence staining (IF).

Tests	IVH(WT)
IF	3

(Notes: Number of mice; Total number: 3.)

Experiment 4: Immunofluorescence staining (IF), Western blots (WB), RT–qPCR, ELISA, NO analysis, hydrocephalus and neurofunction assessment.

Tests	Sham(WT)	IVH(WT)+Vehicle	IVH(WT)+DNase I
IF	6	6	6
WB	6	6	6
RT-qPCR	6	6	6
ELISA	6	6	6
NO	6	6	6
MRI	6	6	6
Open-field test	6	6	6
mNSS	6	6	6
Morris water maze test	6	6	6

(Notes: Number of mice; Total number: 90)

Experiment 5: Meningeal lymphatic vessel drainage function assessment.

Tests	Sham(WT)	IVH(WT)+Vehicle	IVH(WT)+DNase I
IF	18	18	18
Living image	6	6	6
Camera	6	6	6

(Notes: Number of mice; Total number: 90)

Experiment 6: Cell experiment

Tests	Sham(WT)

Neutrophil isolation	18

(Notes: Number of mice; Total number: 18, 18 mice from experiment 4)

Experiment 7: Meningeal lymphatic vessel drainage function assessment

Tests	Sham(WT)	IVH(WT)+Vehicle	IVH(WT)+DNase I
IF	12	12	12
WB	6	6	6
MRI	6	6	6
Open-field test	6	6	6
mNSS	6	6	6

(Notes: Number of mice; Total number: 72)

Experiment 8: Meningeal lymphatic vessel drainage function assessment

Tests	Sham	IVH(WT)	IVH($Cx3cr1^{-/-}$)
WB	6	6	6
IF	6	6	6
MRI	6	6	6
Open-field test	6	6	6

(Notes: Number of mice; Total number: 54)

Experiment 9: Cell experiment

Tests	Sham(WT)
Neutrophil isolation	6

(Notes: Number of mice; Total number: 6, 6 mice from experiment 4.)
